# Role of Nanoscale Inhomogeneities in Co_2_FeO_4_ Catalysts during the Oxygen Evolution Reaction

**DOI:** 10.1021/jacs.2c00850

**Published:** 2022-06-29

**Authors:** Felix Thomas Haase, Anna Rabe, Franz-Philipp Schmidt, Antonia Herzog, Hyo Sang Jeon, Wiebke Frandsen, Praveen Vidusha Narangoda, Ioannis Spanos, Klaus Friedel Ortega, Janis Timoshenko, Thomas Lunkenbein, Malte Behrens, Arno Bergmann, Robert Schlögl, Beatriz Roldan Cuenya

**Affiliations:** † Department of Interface Science, Fritz Haber Institute of the Max Planck Society, 4-6 Faradayweg, Berlin 14195, Germany; ‡ Inorganic Chemistry and Center for Nanointegration Duisburg-Essen (CENIDE), University of Duisburg-Essen, 7 Universitätsstr., Essen 45141, Germany; § Inorganic Chemistry, Christian Albrechts University, 2 Max-Eyth-Straße, Kiel 24118, Germany; ∥ Department of Inorganic Chemistry, Fritz Haber Institute of the Max Planck Society, 4-6 Faradayweg, Berlin 14195, Germany; ⊥ Max Planck Institute for Chemical Energy Conversion, 34-36 Stiftstrasse, Mülheim an der Ruhr 45470, Germany

## Abstract

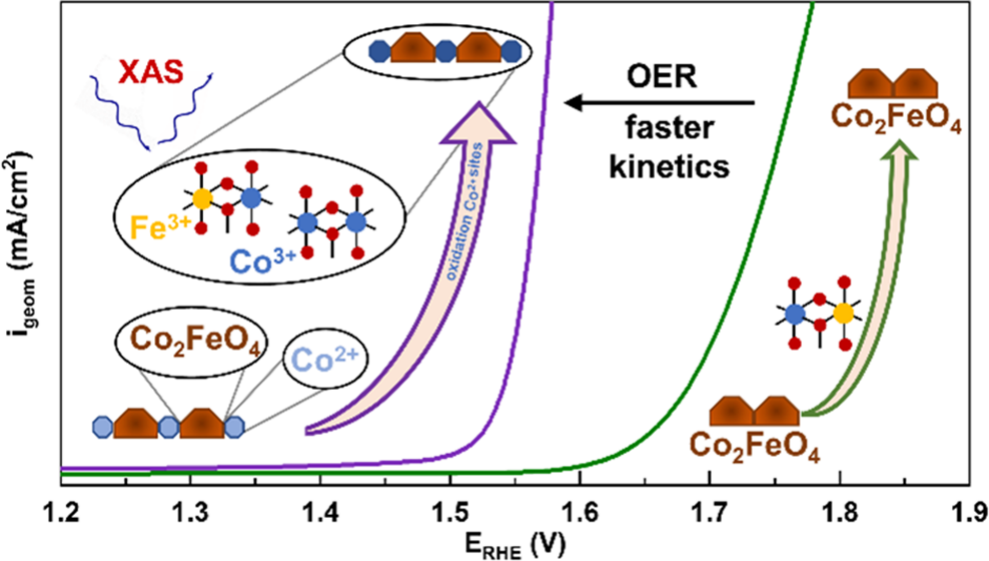

Spinel-type catalysts are promising anode materials for the alkaline oxygen evolution reaction (OER), exhibiting low overpotentials and providing long-term stability. In this study, we compared two structurally equal Co_2_FeO_4_ spinels with nominally identical stoichiometry and substantially different OER activities. In particular, one of the samples, characterized by a metastable precatalyst state, was found to quickly achieve its steady-state optimum operation, while the other, which was initially closer to the ideal crystallographic spinel structure, never reached such a state and required 168 mV higher potential to achieve 1 mA/cm^2^. In addition, the enhanced OER activity was accompanied by a larger resistance to corrosion. More specifically, using various *ex situ*, *quasi in situ*, and *operando* methods, we could identify a correlation between the catalytic activity and compositional inhomogeneities resulting in an X-ray amorphous Co^2+^-rich minority phase linking the crystalline spinel domains in the as-prepared state. *Operando* X-ray absorption spectroscopy revealed that these Co^2+^-rich domains transform during OER to structurally different Co^3+^-rich domains. These domains appear to be crucial for enhancing OER kinetics while exhibiting distinctly different redox properties. Our work emphasizes the necessity of the *operando* methodology to gain fundamental insight into the activity-determining properties of OER catalysts and presents a promising catalyst concept in which a stable, crystalline structure hosts the disordered and active catalyst phase.

## 1 Introduction

Water electrolysis is the most promising approach to produce fossil-fuel-free (green) hydrogen. However, the anodic oxygen evolution reaction (OER) remains the bottleneck due to the involved 4-electron mechanism.^1−5^ The required high overpotentials compromise its application and limit the efficiency of electrolyzers used in combination with electricity from renewable power sources.^6−11^

The implementation of nonprecious and earth-abundant anode materials remains an important aspect. Ir- and Ru-based catalysts excel in terms of the OER activity in acidic electrolytes relevant for membrane-based electrolyzers.^12−14^ However, in alkaline and neutral media, Co-, Ni-, and Fe-oxide catalysts are promising alternatives.^1,15−20^ To enhance knowledge-driven catalyst design, spinel-type catalysts offer significant advantages due to their flexibility in terms of composition, morphology, and their stable crystal structure.^21−24^ The general chemical notation for a spinel is A^2+^B_2_^3+^O_4_^2–^. In a normal spinel, the A^2+^ cations occupy the tetrahedral sites, whereas the B^3+^ ions are located at the octahedral sites, as, for example, in Co_3_O_4_. In an inverse spinel, half of the B^3+^ cations occupy all tetrahedral sites and the remaining B^3+^ and A^2+^ are in octahedral sites, with Fe_3_O_4_ in the form of magnetite being a prominent example. Therefore, the cation substitution of Co with Fe is appealing as it does not only change the chemical composition but also changes the degree of inversion and the magnetic properties.^22,24,25^ Within one chemical composition, the cation site occupation in the O sub-lattice is prone to variations.^26,27^ Recently, we showed that this occupation transforms dynamically in response to an anodic potential in spinel-like Co*_X_*Fe_3_*_–X_*O*_Y_* nanoparticles.^28^ Co- and Fe-based spinel oxides are widely regarded as affordable and stable anode materials and are being considered for industrial applications, with low reported overpotentials.^24,29−33^ The stability of the spinel crystal structure has been underlined by a study of Co_3_O_4_ films, which reversibly form amorphous CoO*_X_*(OH)*_Y_* with more pronounced di-μ-oxo-bridged Co ions under OER conditions but recrystallize to the initial spinel structure after the reaction.^34^ X-ray amorphous domains can be present upon preparation and play a beneficial role in the kinetics in the OER.^35,36^ Furthermore, including Fe into Co- and Ni-oxide catalysts has proven to enhance their OER activity.^19,31,37^ Therein, the importance of Co^3+^ ions in octahedral sites for OER activity has been shown.^38−40^ In terms of the activity, single-particle OER measurements with CoFe_2_O_4_ nanoparticles with sizes below 5 nm exhibited high current density without experiencing irreversible changes in their crystallinity and morphology.^41^ In the case of Co_2_FeO_4_ spinel catalysts, it was suggested that the introduction of Fe^3+^ ions into a Co_3_O_4_ spinel system activates Co^3+^ sites by delocalization of the Co 3d electrons.^42^ The role of Co^3+^ sites was investigated on different Co oxides, where the OER activity was found to increase with the Co^3+^ site reducibility.^43^

In this work, we compare two structurally equal Co_2_FeO_4_ spinels with nominally identical stoichiometry but which exhibit substantially different OER activities. Here, the redox electrochemistry was investigated and compared with results from *quasi in situ* X-ray photoelectron spectroscopy and electron microscopy. In combination with *operando* X-ray absorption spectroscopy, we shed light on the underlying properties responsible for the differences in their catalytic behavior. In particular, we identified a beneficial role of an amorphous minority phase linking the crystalline Co_2_FeO_4_ host structure for enhanced OER activity.

## 2 Experimental Section

### 2.1 Synthesis

The syntheses of the two Co_2_FeO_4_ catalysts were carried out in an automatic lab reactor system (*OptiMax* 1001, *Mettler Toledo*), following a crystalline precursor decomposition approach.

For the aqueous conventional coprecipitation synthesis of the layered double hydroxide (LDH) precursor, 125 mL of a 0.266 M Fe(NO_3_)_3_·9H_2_O and 0.533 M Co(NO_3_)_2_·6H_2_O solution was continuously dosed for an hour into a single-wall glass reactor prefilled with 200 mL of deionized water. The temperature was kept constant at 50 °C, and the pH was controlled by an InLab Semi-Micro-L pH electrode. A pH of 8.5 was guaranteed through the automatic dosing of a precipitating agent, which was a mixture of 0.6 M NaOH and 0.09 M Na_2_CO_3_ solutions. The precipitate was aged for 1 h at 50 °C without further pH control. After cooling to room temperature, the dispersion was washed with deionized water several times and dried in an oven for at least 12 h at 80 °C in air.

The microemulsion-assisted coprecipitation was carried out similar to the procedure described above. The aqueous phases, consisting of the prefilled water, the metal salt solution (0.133 M Fe(NO_3_)_3_·9H_2_O and 0.266 M Co(NO_3_)_2_·6H_2_O solutions), and the precipitation agent (0.15 M NaOH and 0.0225 M Na_2_CO_3_ solutions), were each introduced into water-in-oil microemulsions, containing cyclohexane, Triton X-100, 1-hexanol, and the aqueous phase. The applied formulation results in only 8.4% of aqueous phase by volume. For preparation of the microemulsions, Triton X-100, 1-hexanol, cyclohexane, and the corresponding aqueous phase were mixed and stirred until a clear solution formed. Afterward, the reaction was carried out as described above. To remove the surfactant, the precipitate was washed 5 times with acetone and 10 times with ethanol. Consecutively, the precipitate was dried in an oven for at least 12 h at 80 °C in static air.

The as-prepared LDHs were calcined at 400 °C for 3 h with a heating ramp of 2 K/min in a muffle furnace (*Nabertherm LE* 6/11/*B*150) to obtain the Co_2_FeO_4_ spinels.

### 2.2 X-ray Diffractometry

The X-ray diffraction patterns were recorded with a Bruker D8 Advance using a Cu X-ray source in the Bragg–Brentano configuration with a variable primary divergence slit using an energy-dispersive position-sensitive LynxEye XE-T detector (Bruker). The powder measurements and the quantification of the crystallinity were conducted by mixing a CeO_2_ reference (NIST SRM674b) and our powder sample in a 1:1 mass ratio. After rigorous blending, the mixtures were deposited in a Si low background sample holder. The mass fraction of the X-ray amorphous phase was calculated via Rietveld refinement, in which the zero error, sample displacement, lattice parameters, and size-induced broadening were taken into account. The Rietveld refinement was jointly performed for the diffractograms of the two Co_2_FeO_4_ samples mixed with the CeO_2_ standard as well as for the pure CeO_2_ standard measured alone using the same structural parameters for the CeO_2_ as well as the zero error and the background signals from the sample holder.

To record the diffractograms of Co_2_FeO_4_ before and after OER, the samples were prepared on a carbon foil (0.125 mm, 99.95% purity, GoodFellow) and measured with a Bruker D8 Advance in parallel beam configuration with a Goebel mirror and an equatorial Soller slit (0.3°). The applied electrochemical protocol is described in Section 2.3.

### 2.3 Electrochemical Characterization

All herein mentioned measurements were done in 0.1 M KOH (99.98%, semiconductor grade, Sigma-Aldrich) using a three-electrode setup in an electrochemical PTFE cell (Pine Research). The working electrode was a glassy carbon rotating disk electrode (RDE, Pine Research) with a 5 mm diameter and a 0.196 cm^2^ geometrical surface area embedded in a PEEK holder. For the electrode preparation, 2.5 mg of each catalyst was dispersed in 250 μL of EtOH (Sigma-Aldrich) and 250 μL of H_2_O (Milli-Q, 18.2 MΩ) and consecutively sonicated. The catalyst ink was drop-casted on the glassy carbon disk with a loading of 200 μg/cm^2^. The RDE was driven at 1600 rpm (MSR Rotator, Pine Research). The reference electrode was a single junction Hg/HgO electrode (Pine Research), and the counter electrode was a standard graphite electrode (Pine Research). Prior to all measurements, the reference electrode potential was referenced to the reversible hydrogen electrode (RHE HydroFlex, Gaskatel). The potentiostat was an SP-300 (Biologic). Potentiostatic electrochemical impedance spectroscopy (PEIS) was done to determine the Ohmic resistance. The electrochemically active surface area (ECSA) was determined from PEIS, as described in the literature from double-layer capacitance measurements and normalization with an area-specific capacitance.^18,44,45^ An *R*_u_ + *C*_IL_/*R*_ct_ equivalent electrical circuit with the uncompensated resistance *R*_u_ and the charge-transfer resistance *R*_ct_ was assumed, and the capacitance was retrieved from the Nyquist plot. The capacitance and ECSA for the Co_2_FeO_4_ catalyst were obtained as an average of three individual measurements. The fit was performed with the software EC-Lab (v11.36, Biologic), shown in the Supporting Information Figure S7, and the double-layer capacitance was normalized by a specific capacitance of 40 μF/cm^2^, as suggested for metal oxides at pH 13 to calculate the electrochemical surface area.^18^ The redox electrochemistry was investigated by cyclic voltammograms (CVs) from 1.0 to 1.8 V_RHE_ with a scan rate of 5 mV/s. The catalytic activity was determined by quasi-stationary potential step experiments from 1.48 to 1.8 V_RHE_ with potential steps of 20 mV, which were held for at least 4 min. Each potential step was followed by a PEIS measurement. For all electrochemical and *operando, quasi in situ*, and *ex situ* investigations, the Co_2_FeO_4_ samples were conditioned as described above by 20 CVs from 1.0 to 1.4 V_RHE_ with 50 mV/s. Subsequent linear sweep voltammetry (LSV) with 5 mV/s up to 1.7 V_RHE_ followed by consecutive chronoamperometry for 30 min was done to prompt the OER active state.

### 2.4 Scanning Electron Microscopy Measurements

Scanning electron microscopy (SEM, Hitachi S-4800) measurements were done before and after OER. The catalyst ink was drop-casted on glassy carbon electrodes (SIGRADUR, HTW). The electrochemical procedure was conducted, as explained in Section 2.3. The Hitachi S-4800 was equipped with a cold field emission gun and an energy-dispersive X-ray spectroscopy system (QUANTAX 800, XFLASH6 Detector).

### 2.5 Scanning Transmission Electron Microscopy and Energy-Dispersive X-ray Spectroscopy

Scanning transmission electron microscopy in combination with energy-dispersive X-ray spectroscopy (STEM-EDX) was applied using a ThermoFisher Talos F200x at 200 kV. While scanning the focused electron beam with a semiconvergence angle of 10.5 mrad across the region of interest (100 × 95 nm^2^ and 145 × 105 nm^2^; Figure 4), EDX spectra were acquired at each scanning point by a 4-quadrant detector (Super-X detection system, ThermoFisher). The scanning step size and the acquisition time varied between approximately 100–600 pm and 20–50 μs per pixel, respectively (Figure 4). Multiple frames were acquired, and the collected EDX spectra of each frame were summed up, resulting in an improved signal-to-noise ratio. For quantification of the Fe-to-Co ratio, background-subtracted Fe–K and Co–K lines were considered (using an empirical power law fitting). The peak areas were weighted by the Brown-Powell ionization cross sections, as given within the analysis software (Velox 2.13, ThermoFisher Scientific).

### 2.6 *Quasi In Situ* X-ray Photoelectron Spectroscopy

*Quasi in situ* X-ray photoelectron spectroscopy (*quasi in situ* XPS) links electrochemical measurements with consecutive XPS investigations without exposure to air. The XPS measurements were conducted in an ultrahigh-vacuum (UHV) setup. The X-ray source was a nonmonochromatic Mg anode with 1253.6 eV, which was operated at 250 W. A hemispherical electron analyzer (Phoibos 100, SPECS GmbH) and a pass energy of 15 eV were used with a 54.7° angle between the X-ray source and the analyzer. All measurements were conducted on glassy carbon substrates (SIGRADUR, HTW), and the spectra were aligned to the graphitic carbon peak at a 284.4 eV binding energy.^46,47^ The applied electrochemical protocol is described in the electrochemical section. The PTFE cell was equipped with a Pt counter electrode and a leak-free Ag/AgCl reference electrode (3.4M, eDaq), and the electrochemistry was conducted in an Ar atmosphere. After reaction and while preserving the Ar atmosphere, the Co_2_FeO_4_ samples were carefully rinsed with Ar-purged Milli-Q water to remove the electrolyte from the surface. Analysis of the XPS results was carried out using the Casa XPS software.^48^

### 2.7 Constant Kinetic Energy XPS

Constant kinetic energy XPS measurements were carried out at the ISISS endstation of the BESSY II synchrotron radiation facility at the HZB.^49,50^ All measurements were conducted on glassy carbon substrates, and the spectra were aligned to the valence band measured separately for each excitation energy. The electrochemical protocol for measurements after OER is adopted from Section 2.3. The excitation energy was varied to collect photoelectrons with 550 and 200 eV kinetic energies. The peak areas were normalized by the photon illumination and the photoionization cross sections.^51^

### 2.8 Online ICP-OES

Online inductively coupled plasma optical emission spectrometry (ICP-OES) was conducted to determine the dissolution rate of both, Co and Fe during OER. An electrochemical flow cell with a glassy carbon working electrode area of 0.196 cm^2^ coupled with an ICP-OES (Spectroblue EOP, Ametek) was used.^52^ The catalyst loading was determined to be 200 μg/cm^2^ as for the activity measurements. The electrolyte stream was injected with a flow rate of 0.86 mL/min in a quartz nebulizer operated at an Ar (99.999% purity) flow rate of 0.86 L/min. A background Co and Fe signal 5 min before and after the electrochemical measurements at open-circuit voltage was subtracted from the data during catalysis.

### 2.9 *Operando* X-ray Absorption Spectroscopy

*Operando* X-ray absorption spectroscopy (XAS) measurements were carried out at the CryoEXAFS endstation at the KMC-3 beamline of the BESSY II synchrotron radiation facility at Helmholtz-Zentrum Berlin (HZB). The incident X-ray beam passed through a Si(111) double-crystal monochromator, and the fluorescence signal was recorded with a 13-element Si-drift detector. Reference compounds were measured in transmission mode, where the intensity of the transmitted X-rays was measured by a Si-PIN photodiode. A home-built electrochemical XAS cell was used for all catalytic measurements at the Co–K- and Fe–K-edges for the Co_2_FeO_4_ samples drop-casted on a gas diffusion electrode (GDE, FuelCellStore). Each condition was measured for 30 min for each absorption edge. First, the Co_2_FeO_4_ samples were measured in the dry state as-prepared (ap). In a 0.1 M KOH electrolyte, both Co_2_FeO_4_ were activated by 20 cyclic voltammograms from 1.0 to 1.4 V_RHE_ with a scan rate of 50 mV/s. Subsequent measurements at open-circuit potential (1.0 V_RHE_) followed. Next, o*perando* measurements during OER under applied steady conditions at 1.7 V_RHE_ were performed. Final measurements after OER were conducted at 1.0 V_RHE_ in the electrolyte. XAS data alignment, background subtraction, normalization, and X-ray absorption near-edge structure (XANES) data analysis were carried out using the Athena software.^53^ Extended X-ray absorption fine structure (EXAFS) analysis was conducted by least-squares fitting, as implemented in the FEFFIT code using theoretical photoelectron scattering phases and amplitudes as obtained in FEFF8.5 simulations for reference oxide materials.^54,55^ A list with all fit parameters and details of the applied model is provided in the Supporting Information (SI).

## 3 Results and Discussion

Cobalt iron-layered double hydroxide precursors (LDHs) were synthesized by two different synthesis methods following a conventional coprecipitation in aqueous media and a microemulsion-assisted coprecipitation approach, the latter yielding in a larger specific surface area and distinct pore structure due to the addition of a surfactant. Upon calcination at 400 °C, both materials exhibit a spinel structure as the primary phase, as revealed by powder X-ray diffraction (XRD). These samples are denoted in the following as conventional-Co_2_FeO_4_ and microemulsion-Co_2_FeO_4_, respectively. Rietveld refinements of the diffraction patterns (Table 1 and Figures S1–S3) suggest the presence of a Co-rich and Fe-rich spinel phase.^22,29^ The total crystallinity for both Co_2_FeO_4_ catalysts has been determined via mass fractions based on measurements with an added CeO_2_ reference (NIST SRM674b). A fraction of noncrystalline, amorphous material was obtained for both samples. In particular, 17.6 wt % of the catalysts was found to be X-ray amorphous in the conventional Co_2_FeO_4_ sample, while for the microemulsion Co_2_FeO_4_, this fraction is twice as large, namely, 37.4%. We also calculated the metal–metal distances of di-μ-oxo-bridged metal ions from the Rietveld refinement results, which were subsequently used as the starting point for the EXAFS data fitting. The microemulsion Co_2_FeO_4_ sample exhibits minor phosphate impurities visible in the survey scan of *quasi in situ* XPS data, but a measurement after OER (Figure S4) indicates the complete removal of these species. Since we electrochemically precondition the catalysts before OER, we believe that such a synthesis residue is already removed during the activation treatment and thus cannot affect the electrocatalytic performance of the material.

**Table 1:** Results from the Rietveld Refinement of Powder XRD Pattern with an Added CeO_2_ Reference (NIST SRM674b)^a^

	CeO_2_ standard		Co*_X_*Fe_3*–X*_O_4_ (Fe-rich)		Co_3_*_–X_*Fe*_X_*O_4_ (Co-rich)	
space group	*Fm*3̅*m*		*Fd*3̅*m*		*Fd*3̅*m*	
sample	conv.	micro.	conv.	micro.	conv.	micro.
fraction (wt %)	54.6 ± 10.5	61.51 ± 0.63	37 ± 10	2.1 ± 0.5	8 ± 13	36.4 ± 0.5
crystallite size (nm)	4.1 ± 0.3		6.6 ± 0.4	2.6 ± 0.7	8 ± 3	4.1 ± 0.3
	205.2 ± 1.3					
lattice parameter (Å)	5.3898 ± 0.0019		8.188 ± 0.009	8.24 ± 0.10	8.15 ± 0.3	8.139 ± 0.006
	5.41165 ± 0.00001					

a The Rietveld refinement showed two differently sized CeO_2_ phases, as well as Fe- and Co-rich spinel phases.

A significant difference is the larger Brunauer–Emmett–Teller (BET) surface area of the microemulsion Co_2_FeO_4_ sample, with 153 m^2^/g as compared to 90 ± 1 m^2^/g. The larger physical surface area is as well visible in the SEM images, showing a more mesoporous structure for the microemulsion Co_2_FeO_4_ (Figure S5) as compared to the 700 nm larger flakes in the conventional Co_2_FeO_4_ sample.

**Figure 1:**
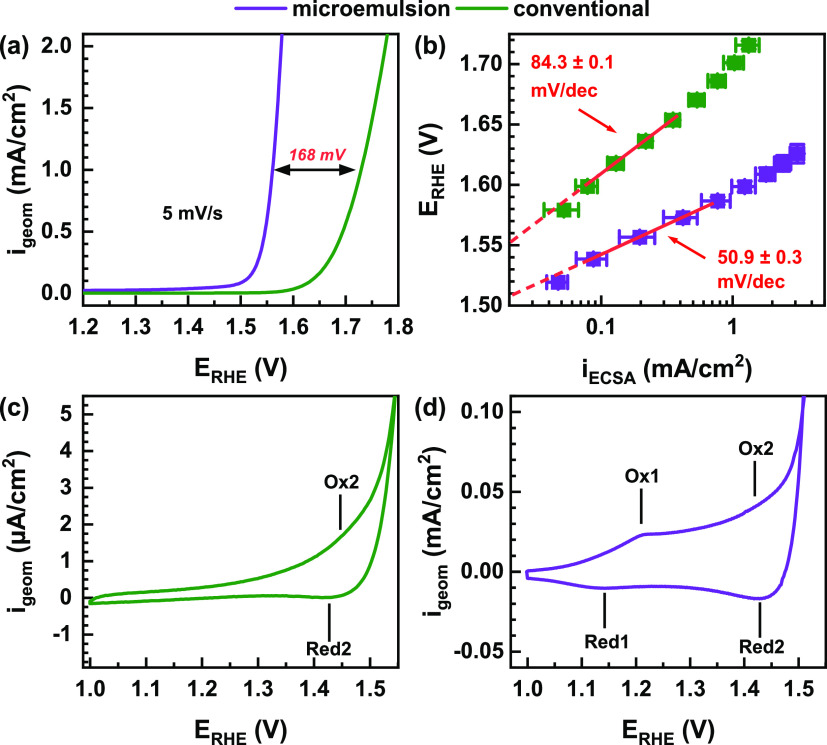
Electrochemical OER characterization in 0.1 M KOH. (a) Electrochemical activity plot with linear sweep voltammetry (LSV, 1.0–1.8 V_RHE_, 5 mV/s) of conventional and microemulsion Co_2_FeO_4_ samples, with the comparison at 1 mA/cm^2^. (b) Tafel plots with current densities normalized by the electrochemical active surface area and derived Tafel slopes. Cyclic voltammetry of conventional (c) and microemulsion (d) Co_2_FeO_4_ samples with a 5 mV/s scan rate from 1 to 1.8 V_RHE_. The positions of the distinct redox transitions are sketched in the diagram.

To evaluate the differences in the electrocatalytic OER activity and in the redox electrochemistry of the above-described samples, we performed comprehensive electrochemical measurements in 0.1 M KOH using a rotating disk electrode (RDE) setup. Linear sweep voltammograms (LSVs) between 1 and 1.8 V_RHE_, displayed in Figure 1a, illustrate the lower overpotential for the microemulsion Co_2_FeO_4_, as compared to the conventional Co_2_FeO_4_ when normalized by the geometric surface area using an identical catalyst loading. At 1 mA/cm^2^, the conventional Co_2_FeO_4_ exhibits a 168 mV higher potential than the microemulsion Co_2_FeO_4_. The electrochemically active surface area (ECSA) was determined through the double-layer capacitance *C*_IL_ retrieved from PEIS measurements.^18,44,45^ The ECSA of the microemulsion Co_2_FeO_4_ is 0.357 ± 0.091 cm^2^, which is in agreement with the BET results, ∼1.5 times larger than that of the conventional Co_2_FeO_4_, with 0.239 ± 0.123 cm^2^.

Figure 1b shows a Tafel plot with the current density normalized by the ECSA. Also, in this case, the microemulsion Co_2_FeO_4_ exhibits significantly higher current densities than the conventional Co_2_FeO_4_, suggesting pronounced differences in their intrinsic catalytic activity. The microemulsion Co_2_FeO_4_ exhibits a notably lower charge-transfer resistance under OER conditions (Figure S8). We furthermore identified a significantly lower Tafel slope for the microemulsion Co_2_FeO_4_ sample, indicating preferable OER kinetics. Differences in Tafel slopes can represent differences in the OER rate-limiting processes and have been previously correlated not only to differences in the near-surface structure, but also to the conductivity of the catalysts.^56^Figure S9 shows the intersection of the extrapolated, linear Tafel regime with a linear fit of the non-OER regime. The point of intersection provides the required minimum potential or onset potential to enter the OER regime following the Butler–Volmer equation, and Faradaic currents at lower potentials are assigned to parasitic, non-OER processes. This potential is 1.567 V_RHE_ for the conventional and 1.520 V_RHE_ for the microemulsion Co_2_FeO_4_ sample. Therefore, the OER onset potential of the conventional Co_2_FeO_4_ catalyst was determined as 47 mV higher than for the microemulsion Co_2_FeO_4_.

Figure 1c,d shows cyclic voltammograms (CVs) with typical features of Co-based electrocatalysts due to redox transitions of the Co–O.^57^ We did not identify any additional Fe-related redox transitions. The CV of the microemulsion Co_2_FeO_4_ shows two broad redox transitions with an oxidation peak (Ox1) at ∼1.2 V_RHE_ and a reduction peak (Red1) at ∼1.15 V_RHE_ as well as (Ox2-Red2) at ∼1.45 V_RHE_. The oxidation peak (Ox2) coincides with the onset of the OER and results from charge redistribution in the Co–O* ligand environment.^5,56^ On the contrary, the conventional Co_2_FeO_4_ exhibits only one broad redox transition, while the redox transition (Ox1, Red1) at lower potential is not visible. These distinctly different ratios of the redox features already suggest structural differences in the composition and nature of the Co–O redox sites, as the pronounced transition at lower potential has been previously predominantly found for layered CoOOH-like structures. In contrast, the redox transition Ox2/Red2 at higher electrode potentials was primarily present in the case of Co_3_O_4_ catalysts.^43^

Thus, we identified differences in the redox electrochemistry as well as in the mass-based and surface area-normalized current densities. Those findings indicate that the two Co_2_FeO_4_ samples differ substantially in their catalytically relevant near-surface redox chemistry and thus in the characteristics of their active catalyst state. As those disparities evidently go beyond plain differences in the available active surface area (Figure 1b), a comprehensive catalyst characterization is required to better understand the activity-determining properties.

First, SEM images after OER (Figure S6) did not indicate pronounced morphological differences in the after-OER state as compared to the as-prepared state. The XRD pattern after OER (Figure S10) still showed the spinel pattern with the (311) Bragg peak at 36.3°, which suggests the structural integrity of the spinel crystallites for conventional Co_2_FeO_4_ but exhibits a broadening after OER for the microemulsion Co_2_FeO_4_.

**Figure 2:**
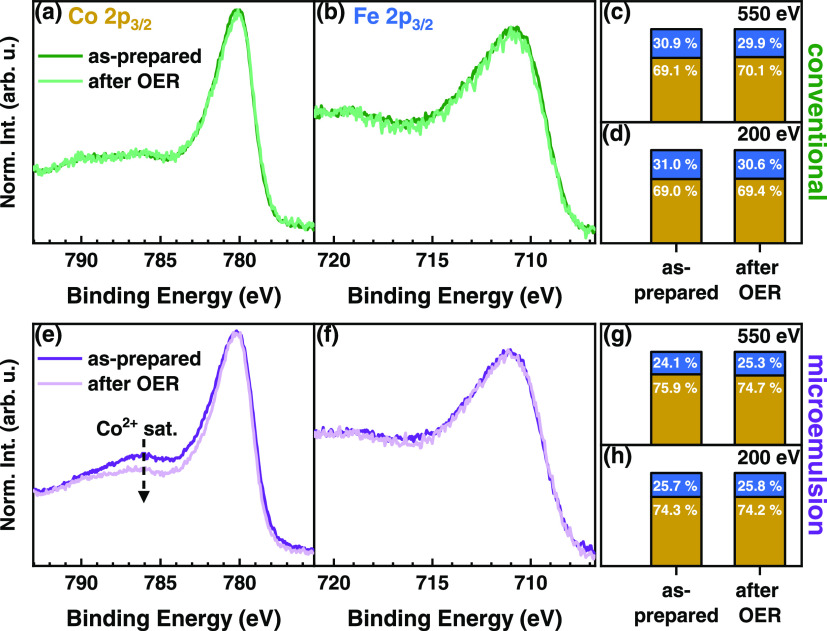
*Quasi in situ* XPS data of conventional and microemulsion Co_2_FeO_4_ before and after OER measured with a Mg X-ray anode. The maximum normalized intensity of Co 2p_3/2_ is shown in panel (a) for the conventional sample and in panel (e) for the microemulsion one. The Fe 2p_3/2_ data for the conventional sample are shown in panel (b) and for the microemulsion in panel (f). Atomic percentages of Co (yellow) and Fe (blue) species from XPS measurements with 200 and 550 eV kinetic photoelectron energies before and after OER are displayed in panels (c, d) and (g, h).

To track the evolution of the near-surface composition and chemistry upon OER, we investigated the catalyst before and after OER with quasi in situ X-ray photoelectron spectroscopy (XPS) setup. Figure 2 shows a qualitative and quantitative comparison of the Co 2p_3/2_ region, and fits of the Co 2p_3/2_, Fe 2p_3/2_, and O 1s regions are shown in Figures S11–S13 of the Supporting Information. The conventional Co_2_FeO_4_ does not exhibit any obvious differences in the Co 2p_3/2_ and Fe 2p_3/2_ regions before and upon OER, revealing a strong integrity of the chemical state of the near-surface metal ions. The Co 2p_3/2_ XPS region shows a striking similarity with the Co_3_O_4_ spinel compounds from the literature, having Co ions distributed among the octahedral (O_h_) and tetrahedral (T_d_) sites.^58,59^ The Fe 2p_3/2_ region, and especially the absence of a shoulder at ∼708 eV, agrees well with a Fe^3+^-rich near-surface, which does not change irreversibly during OER (Figure S12). In contrast, an irreversible reduction of magnetite surfaces during OER was reported for single-crystal studies.^60^ By studying the near-surface oxygen chemistry, we found in the O 1s region (Figure S13) of the conventional Co_2_FeO_4_ sample that the metal–O species dominate, though the fraction of M-OH slightly increases from 9.0% in the as-prepared state to 17.4% after OER.

Nonetheless, we identified a strong change in the near-surface chemical state of the microemulsion Co_2_FeO_4_ sample, especially in the Co 2p_3/2_ satellite feature at 787 eV, which is commonly attributed to Co^2+^.^58,59,61^ This satellite feature is more pronounced in the as-prepared state as compared to the conventional Co_2_FeO_4_ sample, and the fitting shows a decrease of the fraction by ∼15% after OER. After OER, the Co 2p_3/2_ spectrum of the microemulsion Co_2_FeO_4_ sample resembles that of a Co_3_O_4_ spinel. These findings suggest an irreversible oxidation of Co^2+^ to Co^3+^ during the oxidative reaction conditions of oxygen evolution. The Fe 2p_3/2_ region of the microemulsion Co_2_FeO_4_ resembles that of the conventional Co_2_FeO_4_ sample and does not indicate the presence of Fe^2+^. As displayed in Figure S13, the near-surface oxygen spectrum of the microemulsion Co_2_FeO_4_ in the as-prepared state is also dominated by M–O species, but shows a significantly higher fraction of M-OH (27.5%) as compared to the conventional Co_2_FeO_4_ (9.0%) sample. However, the M-OH fraction in the microemulsion Co_2_FeO_4_ decreases to 22.4% after OER, which is in line with the irreversible Co^2+^ oxidation. After OER, surface Co^2+^-rich domains in the microemulsion Co_2_FeO_4_ sample are irreversibly oxidized to Co_3_O_4_, which is reflected in a convergence of the M-OH fraction in the two Co_2_FeO_4_ after OER.

In addition to the near-surface chemistry, the Co_2_FeO_4_ may be prone to compositional changes upon OER. A comparison of the Co:Fe ratio of the two Co_2_FeO_4_ catalysts revealed compositional differences, as displayed in Figure 2. Thus, we performed ex situ depth-dependent XPS measurements with constant kinetic photoelectron energy to compare the Co:Fe ratio for different information depths in the termination layer, as shown in Figure S14. Within the inelastic mean free path of ∼10.4 and ∼5.6 Å, no compositional differences were visible, in contrast to the expectation for a core–shell structure.

**Figure 3:**
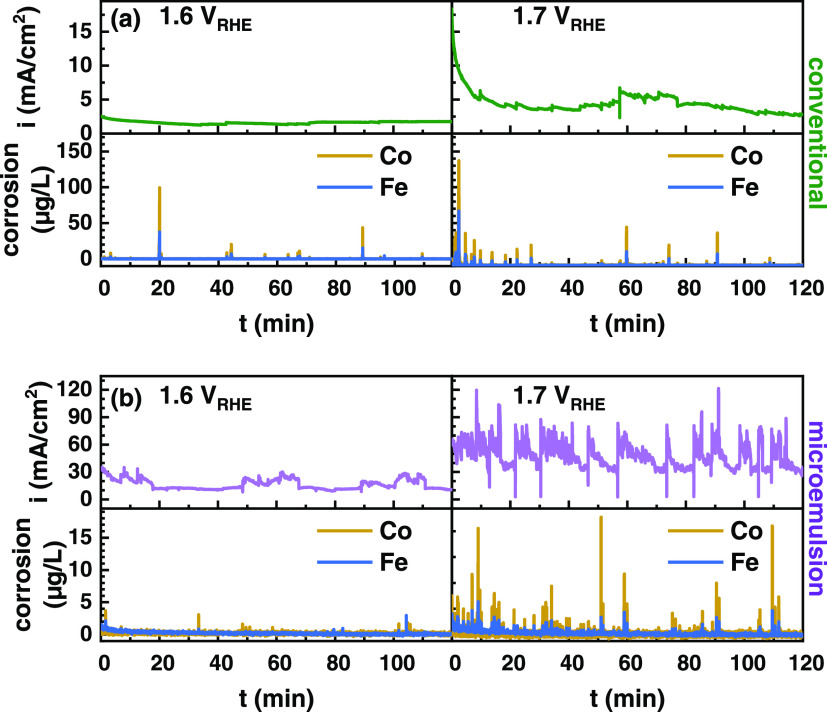
Chronoamperometric flow cell measurements acquired during OER for 2 h for the conventional (a) and microemulsion (b) Co_2_FeO_4_ samples at 1.6 V_RHE_ and 1.7 V_RHE_, respectively. The real-time Co and Fe dissolution was tracked with online inductively coupled plasma optical emission spectroscopy (ICP-OES).

The primary reason for compositional changes in the termination layer is electrocatalytically induced dissolution, as reported for Fe sites in Fe–MO_X_H catalysts.^62^ Therefore, we studied the compositional stability of the Co_2_FeO_4_ electrocatalysts by flow cell–electrochemical online inductively coupled plasma optical emission spectrometry (ICP-OES) experiments. We tracked the corrosion of Co and Fe during OER at 1.6 and 1.7 V_RHE_ for both catalysts for 120 min, which is preceded by 5 min at OCV and the electrochemical activation (Figure 3 and Table 2). Notably, the dissolution rate at OCV is negligible (Figure S15). During OER, the conventional Co_2_FeO_4_ exhibits a constant Co:Fe dissolution ratio of 2.4, while the microemulsion catalyst dissolves Co and Fe with a ratio of 3.5 and 1.6 at 1.6 V_RHE_ and 1.7 V_RHE_, respectively. Thus, the dissolution rate of the conventional Co_2_FeO_4_ is more similar to the nominal composition, while the potential dependence for the microemulsion Co_2_FeO_4_ suggests that the dissolution rather stems from the Co-rich minority phase, which appears to become stabilized upon conditioning at 1.6 V_RHE_. The increased stability and a certain heterogeneity in the termination layer of the microemulsion catalyst are given.

**Table 2:** Dissolution of Co and Fe during 2 h of OER at 1.6 V_RHE_ and 1.7 V_RHE_

measurement		Co dissolution rate (ng/min)	Fe dissolution rate (ng/min)	Co:Fe ratio	Co loss after 2 h (%)	Fe loss after 2 h (%)
1.6 V_RHE_	conventional	5.65	2.35	2.4	2.7	2.1
	microemulsion	2.05	0.59	3.5	1.2	0.6
1.7 V_RHE_	conventional	13.5	5.6	2.4	6.5	5.0
	microemulsion	6.51	4.18	1.6	3.9	4.1

**Figure 4:**
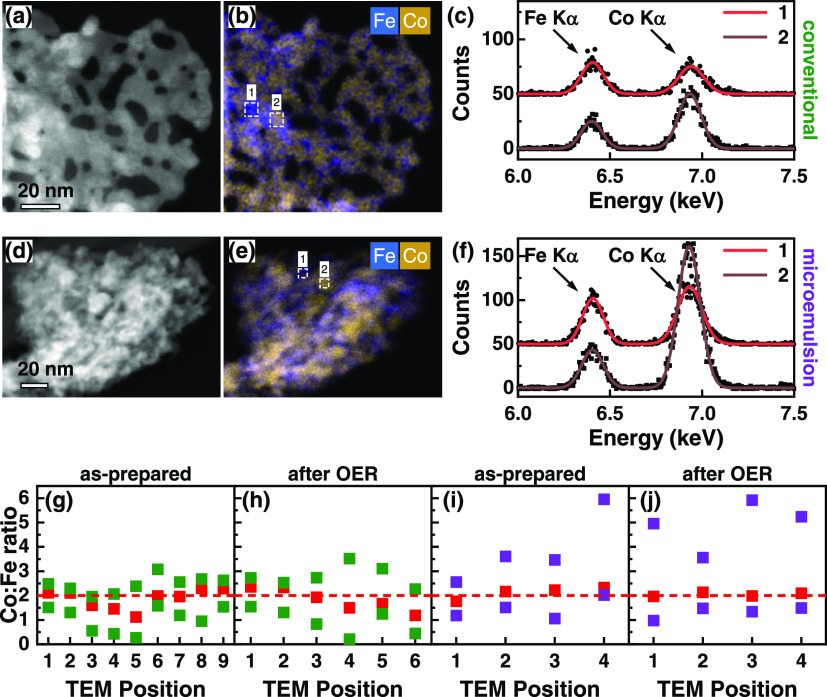
Representative images of conventional vs microemulsion as-prepared Co_2_FeO_4_ samples. (a) STEM dark-field image of the conventionally prepared Co_2_FeO_4_. (b) EDX map, comparing the elemental distribution of Fe (blue) and Co (yellow). The white dashed rectangles highlight 6 × 6 nm^2^ areas with increased Fe (1) or slightly increased Co (2) content with respect to the nominal atomic ratio of Co:Fe = 2. (c) EDX spectra extracted from the two regions 1 and 2 shown in panel b, depicting the different Fe-to-Co peak ratios. (d)–(f) Same as in panels (a–c) but for the microemulsion Co_2_FeO_4_. The spectra in panel (f) show again (1) Fe- and (2) Co-enriched areas. The Co enrichment is much stronger compared to the conventional Co_2_FeO_4_ sample. (g)–(j) Comparison of the Co:Fe ratio at different locations (5–20 nm large scanning regions) in the conventional and microemulsion samples. The Co:Fe ratio of areas larger than 300 × 300 nm^2^ is denoted with a dashed red line. The TEM positions reflect different crystallites from arbitrary positions of the TEM grid. Within each TEM position, regions of the highest and lowest Co:Fe ratios are shown together with the average value as a red data point.

To extract more local information on the morphological and compositional evolution of the Co_2_FeO_4_ as well as possible heterogeneities, we performed STEM-EDX investigations before and after OER. Figure 4 shows a comparison between the as-prepared conventional and microemulsion Co_2_FeO_4_ on the local scale. The conventional Co_2_FeO_4_ catalyst consists of ensembles of *sub*-10 nm domains forming networks with 5–10 nm pores (Figure 4a). The EDX map in Figure 4b displays the elemental distribution of Fe (blue) and Co (yellow). From that, we found local variations in the Co:Fe ratio with higher or lower Co content with respect to the nominal Co:Fe ratio value of 2 (and vice versa for Fe). To corroborate these findings, we extracted local EDX spectra from the image shown in Figure 4c, according to 6 × 6 nm^2^ areas highlighted by the white dashed rectangles 1 and 2 in Figure 4b. The lower spectrum shows a Co-rich region with a slightly increased Co content (Co:Fe = 2.18), while the upper spectrum has a clear drop in the Co Kα peak and an increased Fe Kα peak, resulting in an Fe-rich region (Co:Fe = 0.92). The average ratio of the whole position in (b) is 1.99, which agrees very well with the nominal and XPS-based Co:Fe ratio. The microemulsion Co_2_FeO_4_, in contrast, exhibits smaller oxide domains forming a foam-like structure with less clear porosity, presumably due to a smaller pore size (Figure 4d). An EDX map (e) and local spectra from regions 1 and 2 (f) exhibit again local variations in the Co:Fe ratio in the same size range as those observed for the conventional Co_2_FeO_4_. Regions 1 and 2 (white rectangles in (e), 6 × 6 nm^2^) reveal again Co-rich (Co:Fe = 3.77) and Fe-rich (Co:Fe = 1.37) areas. Importantly, the local Co enrichment is significantly higher than for the conventional Co_2_FeO_4_ sample.

As the elemental distribution may vary not only on the very local scale (5–10 nm) but also on a larger scale within the same sample, we repeated the STEM-EDX measurements on different regions of crystallites from arbitrarily chosen positions on the TEM grid. This was done for all four samples, the conventional and microemulsion Co_2_FeO_4_, each before and after OER (Figure 4g–j). First, no significant changes were found in the Co:Fe ratios before and after OER by comparing (g) and (h) for the conventional and (i) and (j) for the microemulsion Co_2_FeO_4_. Second, we identified local variations in the Co:Fe ratio for all samples and different sample positions, including Co-enriched and Fe-enriched regions. Moreover, we observed a stronger local Co enrichment in the microemulsion sample as compared to that in the conventional sample (compare the green points above the red line in (g) + (h) with the purple points above the red line in (i) + (j)).

However, the average Co:Fe ratios (over areas of approximately 300 × 300 nm^2^ or more) remain ∼2 for both samples (red dashed lines in (g)–(j)). For the conventional Co_2_FeO_4_, the local Co:Fe ratio varies from 0.4 up to 2.8. The microemulsion Co_2_FeO_4_ reveals sub-10 nm regions with very high Co concentration, which exceeds the nominal ratio up to 3 times. For example, TEM position 4 in Figure 4i shows a local Co:Fe ratio in the as-prepared microemulsion Co_2_FeO_4_ of 6. The same applies to the microemulsion sample after OER in Figure 4j, with a Co:Fe ratio of also up to 6. Although other Co-enriched regions in the microemulsion Co_2_FeO_4_ (before and after OER) show a lower Co:Fe ratio, in average, the local Co enrichment from sub-10 nm inhomogeneities is significantly higher in this sample versus the conventional Co_2_FeO_4_. Notably, the Co:Fe ratio is not influenced by OER and is a stable characteristic in both samples with a higher Co:Fe ratio variation in the microemulsion Co_2_FeO_4_. Thus, we attribute the near-surface Co enrichment of the microemulsion sample as compared to the conventional Co_2_FeO_4_ determined by XPS to the apparent compositional inhomogeneity revealed by STEM-EDX, which results in less crystallinity and the presence of an amorphous Co-rich secondary phase.

**Figure 5:**
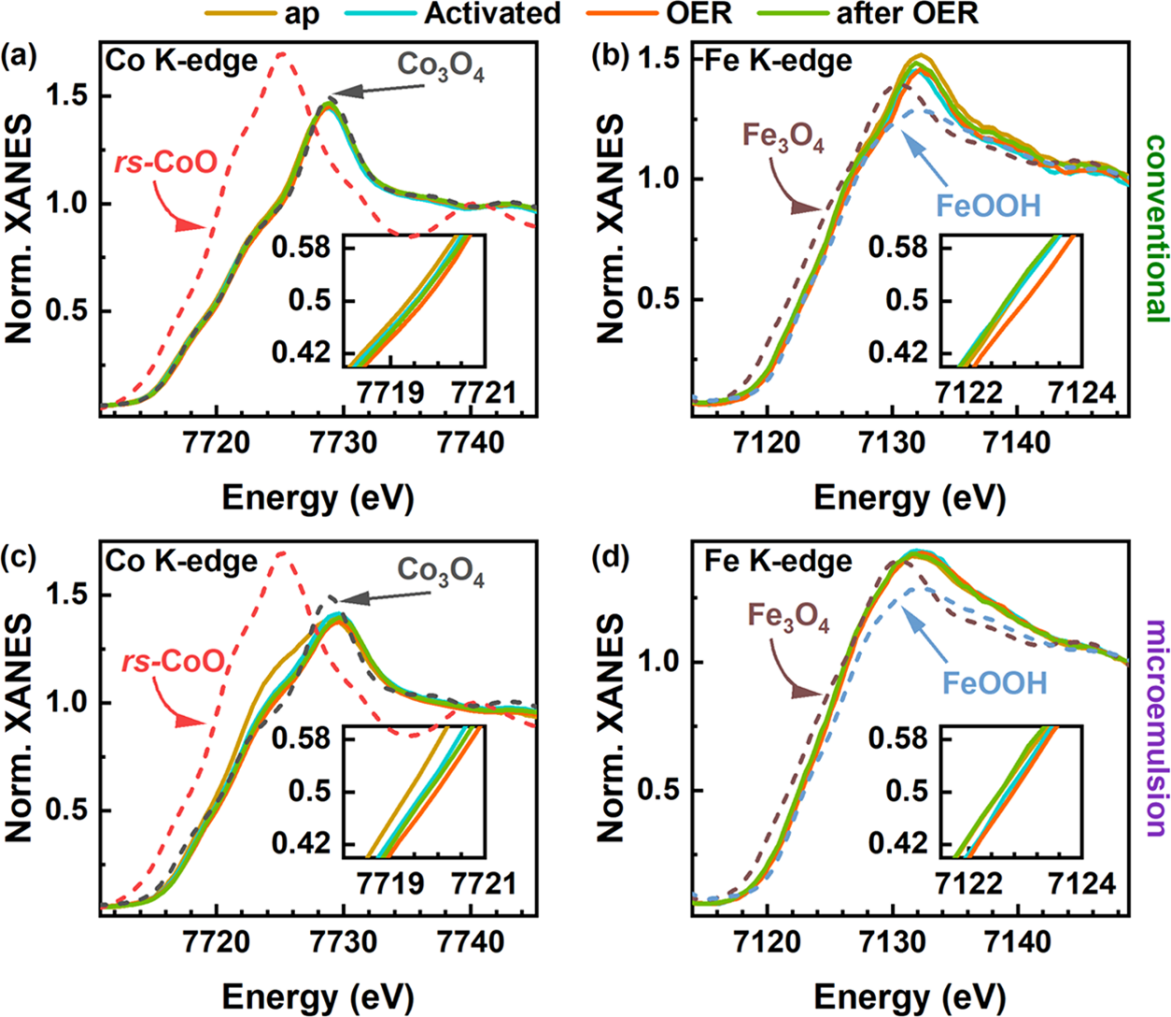
Co K- and Fe K-edge XANES spectra of conventional (a,b) and microemulsion (c,d) Co_2_FeO_4_ with reference spectra for rock salts CoO, Co_3_O_4_, Fe_3_O_4_, and FeOOH. The spectra are displayed for as-prepared (ap) after electrochemical conditioning (activated) at 1 V_RHE_, at 1.7 V_RHE_ (OER), and at an open-circuit potential of ∼1 V_RHE_ after OER. Zoomed-in near-edge regions of X-ray absorption spectra are displayed in the insets to better show the shifts of the absorption edge. Each condition was measured for 30 min per edge.

To track the chemical state and structural evolution of the Co_2_FeO_4_ under reaction conditions, we conducted *operando* X-ray absorption spectroscopy (XAS) measurements. Figure 5 displays the X-ray absorption near-edge structure (XANES) at the Co K- and Fe K-edgesfor the as-prepared state (ap), after electrochemical conditioning (activated), during OER at 1.7 V_RHE_ (OER), and after OER at an open-circuit potential of ∼1 V_RHE_. When compared to the Co_3_O_4_ and Fe_3_O_4_ reference spectra (Figure S16), the Co K- and Fe K-edge XANES in Figure 5a,b of the conventional Co_2_FeO_4_ sample exhibit characteristic features at 7723 eV and 7126 eV, respectively, indicating a spinel-like structure of our sample. In Figure 5c, the microemulsion Co_2_FeO_4_ our sample. In Figure 5c, the microemulsion Co_2_FeO_4_exhibits a noticeable feature at 7722 eV at the Co K-edge in the as-prepared state, which is not observed in the reference spectrum for Co_3_O_4_ spinel. A comparison by linear combination analysis (Figure S17) of the as-prepared Co K-edge spectra showed contributions of 86% Co_3_O_4_ and 14% CoO for the conventional sample but 74% Co_3_O_4_, 18% CoO, and 8% Co(OH)_2_ for the microemulsion Co_2_FeO_4_. The position of the Fe K-edge of the as-prepared microemulsion Co_2_FeO_4_ in Figure 5d agrees well with a Fe^3+^-containing oxide, but its shape neither resembles a specific Fe-oxide reference spectrum nor can it be fitted reasonably by a linear combination of available reference spectra. After activation, the Co K-edge of the conventional and microemulsion Co_2_FeO_4_ samples is shifted to higher energies (the insets of Figure 5). Thereby, the overall shape of the Co K-edge XANES features for the conventional Co_2_FeO_4_ sample did not change, whereas for the microemulsion Co_2_FeO_4_, the feature at 7722 eV diminishes. Thereby, upon activation, the Co K-edge XANES profiles of both Co_2_FeO_4_ samples converged. During OER, the Co edge shifts even further to higher energies and the white line intensity at ∼7730 eV decreases slightly. After OER, the XANES profiles resemble the state before OER, suggesting a reversible active state formation. At this stage, the change in the Co K-edge XANES shape observed during the activation of the microemulsion Co_2_FeO_4_ is, in turn, irreversible. Both samples do not show changes in the shape of the Fe K-edge XANES during the reaction. In particular, the spinel feature at 7126 eV remains more pronounced for the conventional Co_2_FeO_4_. However, for both samples, the Fe K-edge XANES whiteline peak reversibly shifts to higher energies during OER, i.e., shows a qualitatively similar change to that observed at the Co K-edge.

The average Co and Fe oxidation states are calculated using the integral method,^63,64^ which takes both the position and shape of the absorption edges into account calibrated using the Co- and Fe-oxide reference spectra (Figures S18 and S19). The conventional Co_2_FeO_4_ exhibits an average Co oxidation state of 2.67 ± 0.09 and an Fe oxidation state of 2.9 ± 0.2. The microemulsion Co_2_FeO_4_ sample exhibits a lower Co oxidation state with 2.58 ± 0.09 and a similar Fe oxidation state with 2.9 ± 0.2. The lower Co oxidation state can be correlated with the feature at 7722 eV visible in the Co K-edge XANES, where the comparison with CoO and Co(OH)_2_ reference spectra suggests the presence of additional octahedrally coordinated Co^2+^ in the microemulsion sample (Figure S16). After activation, the metal ions oxidize in both Co_2_FeO_4_ samples, exhibiting similar average oxidation states for Co (2.79 ± 0.09) and Fe (2.9 ± 0.2 for the conventional sample, and 3.0 ± 0.2 for the microemulsion Co_2_FeO_4_).

During OER, the Co K-edge position in the microemulsion Co_2_FeO_4_ would correspond to a formal oxidation state of 2.99 ± 0.09 as compared to 2.88 ± 0.09 for the conventional Co_2_FeO_4_. Nonetheless, under OER conditions, we must consider charge reorganization in the Co–O* ligand system, which restricts an absolute determination of the metal oxidation state. However, despite its lower apparent initial oxidation state, the microemulsion Co_2_FeO_4_ oxidizes higher than the conventional Co_2_FeO_4_. For both metal ions and Co_2_FeO_4_, the active state formation is reversible with respect to the formal oxidation state as it decreases after the OER. Thereby, the operando measurements during OER show the reversible oxidation of the catalysts from the perspective of the metal ions. This contrasts the irreversible change in the chemical state observed via XAS after the electrochemical conditioning procedure in the activated state for both Co and Fe. This irreversible change is also evident from the quasi in situ XPS measurements after OER. Notably, the reversible oxidation during OER is more pronounced for the more active microemulsion Co_2_FeO_4_ sample. The changes in the Fe oxidation during OER, in turn, are below the uncertainty of our analysis.

The differences in the redox chemistry of Co and Fe and in the active state formation suggest the differences in the local atomic structure. The *operando* Fourier-transformed extended X-ray absorption fine structure (FT-EXAFS) spectra of both Co_2_FeO_4_ have been analyzed at both K-edges to track changes in the coordination shells of the Co and Fe ions (Figures S20–S22). Following the Rietveld refinement results, we jointly fitted the Co and Fe EXAFS spectra using a spinel model, which quantifies the coordination numbers of both Co_2_FeO_4_ catalysts. We used the metal–metal distances and the total crystallinity as a starting point, and a detailed description of the fitting model can be found in the Supporting Information. Based on the microscopy and spectroscopy results, we considered an amorphous minority phase that predominantly consists of Co^2+^ in addition to the spinel phase. Due to the calcination at 400 °C, we assume an octahedral Co–O coordination for this amorphous Co^2+^ phase.^65^

The contribution of the first-coordination shell, resulting in a peak in the Fourier-transformed (FT) EXAFS at ∼1.5 Å (phase-uncorrected), was approximated with a single Co–O or Fe–O path. The second FT-EXAFS peak at ∼2.6 Å corresponds to the second-coordination shell (Co–M_1_ and Fe–M_1_ paths, where M is Co or Fe) and originates from the di-μ-oxo-bridged backscattering ions. Contributions from mono-μ-oxo-bridged metal ions (Co–M_2_ and Fe–M_2_) in tetrahedral and octahedral sites can be seen at ∼3 Å. During EXAFS fitting, the coordination numbers (CNs) corresponding to atomic pairs in the spinel phase were all linked to a single fitting variable describing the occupancy of the octahedral and tetrahedral sites by Co and Fe ions. We additionally fitted the interatomic distances, disorder factors, and shifts in the reference energy (*E*_0_). The interatomic distances between di-μ-oxo-bridged Co–metal ions from the Rietveld refinement (Table 1) were used as the initial parameter. The EXAFS fitting of the more crystalline conventional Co_2_FeO_4_ sample as-prepared gives a Co–metal distance of 2.870 ± 0.005 Å, similar to the 2.882 Å from the Rietveld refinement for the predominant Co-rich phase. This differs notably for the microemulsion Co_2_FeO_4_ sample, where the Co–metal di-μ-oxo bonds with 2.860 ± 0.005 Å, as obtained from EXAFS data fitting, are significantly shorter than the proposed 2.878 Å from the Rietveld refinement. We attribute this to a contribution of the shorter Co–metal distance of the X-ray amorphous Co^2+^ secondary phase like Co(OH)_2_.

**Figure 6:**
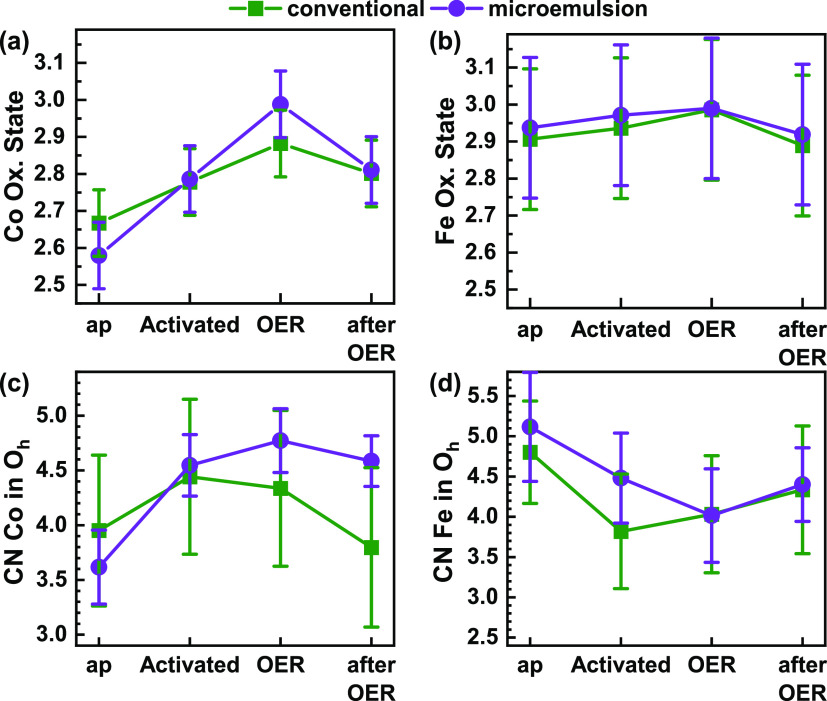
Evolution of the average Co (a) and Fe (b) oxidation states of the conventional and microemulsion Co_2_FeO_4_ catalysts. (c) Co–metal and (d) Fe–metal coordination numbers (CNs) in octahedral sites in the spinel structure under reaction conditions.

The coordination numbers of Co and Fe in octahedral sites in the spinel structure from EXAFS fitting are shown in Figure 6c,d. In the as-prepared state, both Co_2_FeO_4_ catalysts have the same fraction of Co and Fe in octahedral sites. There are more octahedrally coordinated Co and more tetrahedrally coordinated Fe for both catalysts after activation. During OER, the fraction of octahedrally coordinated Co increases further for the microemulsion Co_2_FeO_4_ sample, which contrasts with the evolution of the conventional Co_2_FeO_4_ in which the fraction of octahedrally coordinated Co did not change. After OER, the occupancy of the octahedral sites with Co ions decreases for both Co_2_FeO_4_ catalysts as compared with the states after activation and during OER. Notably, the microemulsion Co_2_FeO_4_ after OER is similar to the activated state, whereas the conventional Co_2_FeO_4_ resembles more the as-prepared state in terms of Co and Fe coordinations. We note that these changes are in agreement with the identified increase in the average Co oxidation state after the activation and during OER. The oxidation of Co and the preference of the octahedral site occupation are reversible processes, similar to what has been suggested for Co_3_O_4_.^34^

This can be explained by an oxidation of the Co^2+^ to Co^3+^ in the activated state and further charge transfer during OER, which is accompanied by a restructuring of the spinel toward an oxyhydroxide phase consisting of primarily di-μ-oxo-bridged metal ions. The correlation of the oxidation state with site occupancy suggests the preference for Co^3+^ in octahedral sites during OER.^38−40,42,66,67^ This concept fits very well with the stronger occupation of the tetrahedral sites by the Co ions after OER at non-OER conditions.

Linking our comprehensive findings on the structure, composition, and chemical state, we emphasize the critical role of the Co-rich domains accompanied by Co^2+^ in the secondary phase, which distinguishes the microemulsion from the conventional Co_2_FeO_4_ sample, as this catalyst exhibits significantly faster kinetics. It was identified as a near-surface Co^2+^ species from the XPS spectrum (Figure 2d) and from the XANES profile (Figure 5c), with a more pronounced metal–hydroxide contribution (Figure S13) consequentially assigned to the amorphous secondary phase linking the spinel domains. We emphasize here the concurrency of the deviations in the near-surface chemical state of Co and O with the presence of highly Co-rich domains (Figure 5). Therefore, the minority phase can be best described as a mixed CoO/Co(OH)_2_-like phase, likely also containing Fe^3+^. Those species oxidize irreversibly during OER and form Co^3+^-rich (oxyhydr)oxide structures, as seen in the (stronger) increase of the average activated Co oxidation state and of the contribution of octahedrally coordinated Co^3+^ (Figure 6). Comparing the redox electrochemistry (Figure 1), we link the pronounced Co^2+/3+^ transitions, representing reducible Co^3+^ sites, to the initially Co^2+^ sites on the Co-rich domains in the as-prepared sample. Considering the properties of the catalytically active state of the Co_2_FeO_4_, we note that the more pronounced Co-related structural and chemical state changes suggest a significantly higher density of redox-active Co ions in the more active microemulsion Co_2_FeO_4_. Following the current state of knowledge, we propose that both, the conventional and the microemulsion Co_2_FeO_4_ samples mainly consist of a spinel host phase, yet the microemulsion Co_2_FeO_4_ holds a linking amorphous phase between crystalline spinel domains, enabling an interplay with likely mobile Co and Fe ions on the surface. This also leads to the formation of a CoO_x_(OH)_y_ adaptation layer during OER on the initially Co^2+^-containing minority phase as well as Co_2_FeO_4_ domains induced from Co precipitation from the soluble amorphous Co sites as followed by ICP-OES.^34,68,69^ Although we showed a reversible oxidation of the Co sites, the low surface-to-volume ratio compared to, e.g., electrodeposited metal (oxy)hydroxide films, limits the extent of the Co edge shift and complementary *operando* O K-edge measurements could provide information on the electronic state of the O-ligand during OER.

Overall, we therefore reveal that more abundant reducible Co^3+^ sites in the vicinity of the Co–Fe spinel host play a key role in the OER catalysis, making the microemulsion Co_2_FeO_4_ a significantly better electrocatalyst. The inherent nanoscale heterogeneity of the microemulsion Co_2_FeO_4_ seems to be beneficial for the kinetics.^70^ Nonetheless, and although Co seems to be critical for the OER activity in Co_2_FeO_4_, we cannot exclude a beneficial effect of mobile Fe ions interacting with the Co-rich minority phase.^37,62^ However, it is evident from our data that the Fe-richer near-surface of the conventional Co_2_FeO_4_ sample alone does not yield in higher catalytic activity, which suggests a threshold in the optimal Co:Fe ratio. In both samples, Fe does not decisively respond to the OER conditions, which suggests low reactivity of the Fe from the host material. Moreover, we emphasize the importance of combining near-surface characterization with advanced electron microscopy to identify compositional nanoscale inhomogeneities that can be linked to the enhanced catalytic properties of heterogeneous powder catalysts being at first glance overall structurally and compositionally very similar. Finally, we also emphasize that the more active microemulsion Co_2_FeO_4_ also excels with a higher corrosion resistance as compared to the conventional Co_2_FeO_4_ sample. This contradicts the commonly identified activity-instability relations, and we assign this to nanoscale heterogeneities in which the structurally flexible Co-rich oxyhydroxide stabilizes the Co_2_FeO_4_ host structure. This finding now allows us to establish new and innovative design concepts for advanced OER catalysts by intentionally introducing secondary amorphous oxyhydroxide phases in crystalline host structures.

## 4 Conclusions

Two stoichiometrically identical and X-ray diffraction-indistinguishable Co_2_FeO_4_ catalysts, synthesized using two different approaches, were found to exhibit drastically different kinetics for the OER. In particular, the microemulsion sample, which was characterized by a metastable precatalyst state, reached much faster the steady-state operation, while the conventionally synthesized sample, with an initial overall crystallinity closer to an ideal spinel Co_2_FeO_4_, required a 168 mV higher potential to reach 1 mA/cm^2^ and never achieved the optimum OER operation state. Moreover, our study demonstrates that detecting local differences in structurally and chemically similar catalysts is crucial to understand catalytically relevant systems.

Comprehensive characterization before and after OER suggests that our Co_2_FeO_4_ catalysts are morphologically, structurally, and compositionally stable as shown by *ex situ* SEM/TEM and exhibit a stable spinel phase visible in *ex situ* XRD as well as online ICP-OES. Nonetheless, the detailed TEM characterization revealed nanoscale inhomogeneities, which would explain the larger Co:Fe ratio in XPS with a Co^2+^- and hydroxide-rich minority phase linking Co_2_FeO_4_ spinel domains and we suggest this to be the reason for the enhanced catalytic activity. Those domains also lead to a lower average apparent Co oxidation state, which irreversibly increases upon OER catalysis, while the heterogeneity of the composition persists. We link the faster kinetics observed for the Co_2_FeO_4_ sample prepared by the microemulsion method to the presence of Co^2+^-rich domains, accompanied by reducible Co^3+^ sites, which are scarce in the less active conventional Co_2_FeO_4_. Our study furthermore shows a correlation between the enhanced presence of octahedrally coordinated Co^3+^ sites (during OER) from the formerly Co^2+^ secondary phase and the distinct redox electrochemistry and enhanced OER catalysis. We also emphasize that Fe abundance in the near-surface itself does not necessarily yield in a highly active catalyst, as the Fe component of this catalyst remained unchanged, suggesting an optimal Co:Fe ratio. Under electrocatalytic conditions, we could link the irreversible transformation in the Co oxidation to the electrochemical activation protocol, but revealed reversible redox dynamics of the Co sites during OER from *operando* XAS data. Both Co_2_FeO_4_ samples exhibit similar structural transformations under OER as the Co ions prefer octahedral sites as a consequence of their oxidation. We explain this with an MO*_x_*(OH)*_y_* termination layer, which forms on both the Co_2_FeO_4_ and the Co-rich domains. Furthermore, the deviations in the nanoscale composition and metal chemical state evidently reduce the corrosion, despite increasing the OER activity. Finally, we would like to highlight the importance of complementary characterization techniques (surface/bulk or local/averaging) to reveal the local chemical state, compositional and structural inhomogeneities of functional heterogeneous catalysts, and their evolution under electrochemical reaction conditions. Only such in-depth insight can provide the much-needed understanding of structure–function correlations in complex heterogeneous catalysts.

## Supporting Information Available

XRD pattern with the Rietveld refinement; SEM images; additional electrochemical analysis; additional Co 2p, Fe 2p, and O 1s XPS spectra and tabulated fitting parameters; and additional XANEX and FT-EXAFS spectra with a detailed explanation of the fitting model and tabulated fitting parameters (PDF)
