# Supplementary material for: Role of Nanoscale Inhomogeneities in Co2FeO4 Catalysts during the Oxygen Evolution Reaction
Source: J Am Chem Soc. 2022 Jun 29;144(27):12007–19. doi: 10.1021/jacs.2c00850 (PMC9284556; doi:10.1021/jacs.2c00850)
Supplement: Supplementary file 1 — ja2c00850_si_001.pdf [file ja2c00850_si_001.pdf]

Supplementary Information:

Role of Nanoscale Inhomogeneities in Co_2FeO_4 Catalysts during Oxygen Evolution Reaction

Felix Thomas Haase[a], Anna Rabe[b,c], Franz-Philipp Schmidt[d,e], Antonia Herzog[a], Hyo Sang Jeon[a], Wiebke Frandsen[a], Praveen Vidusha Narangoda[e], Ioannis Spanos[e], Klaus Friedel Ortega[b], Janis Timoshenko[a], Thomas Lunkenbein[d], Malte Behrens[b,c], Arno Bergmann[a], Robert Schlögl[d,e] and Beatriz Roldan Cuenya[a*]

[a]Department of Interface Science, Fritz Haber Institute of the Max Planck Society, 4-6 Faradayweg, Berlin, 14195, Germany

[b]Inorganic Chemistry and Center for Nanointegration Duisburg-Essen (CENIDE), University of Duisburg-Essen,7 Universitätsstr., Essen, 45141, Germany

[c]Inorganic Chemistry, Christian Albrechts University, 2 Max-Eyth-Straße, Kiel, 24118, Germany

[d]Department of Inorganic Chemistry, Fritz Haber Institute of the Max Planck Society, 4-6 Faradayweg, Berlin, 14195, Germany

[e]Max Planck Institute for Chemical Energy Conversion, 34-36 Stiftstrasse, Mülheim an der Ruhr, 45470, Germany

*Email: roldan@fhi-berlin.mpg.de

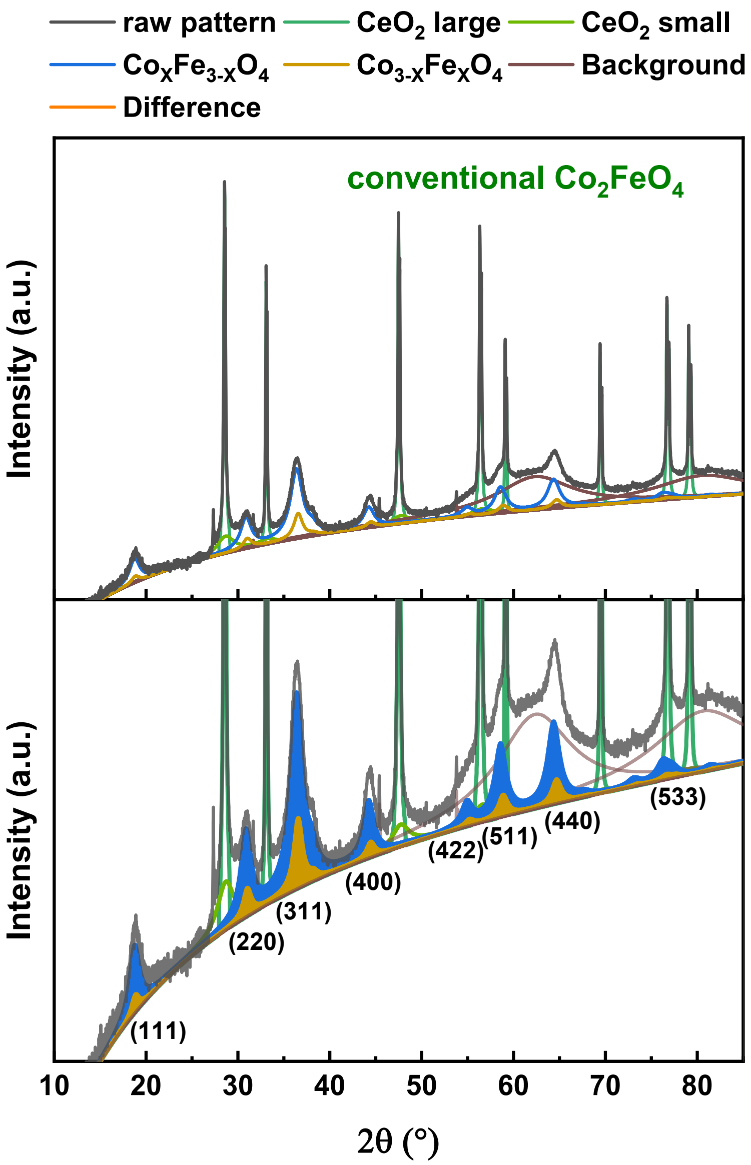

Figure S1. XRD pattern of conventional Co_2FeO_4 with CeO_2 NIST-standard and Rietveld refinement. The signal is displayed in black, CeO_2 standard signal from a larger and smaller crystallite is shown in darker green and lime, underlying background signal from the Si substrate is given in brown. The Rietveld refinement was performed considering an Fe-rich spinel phase (Spinel Phase 1) in yellow and a Co-rich spinel phase (Spinel Phase 2) in blue.

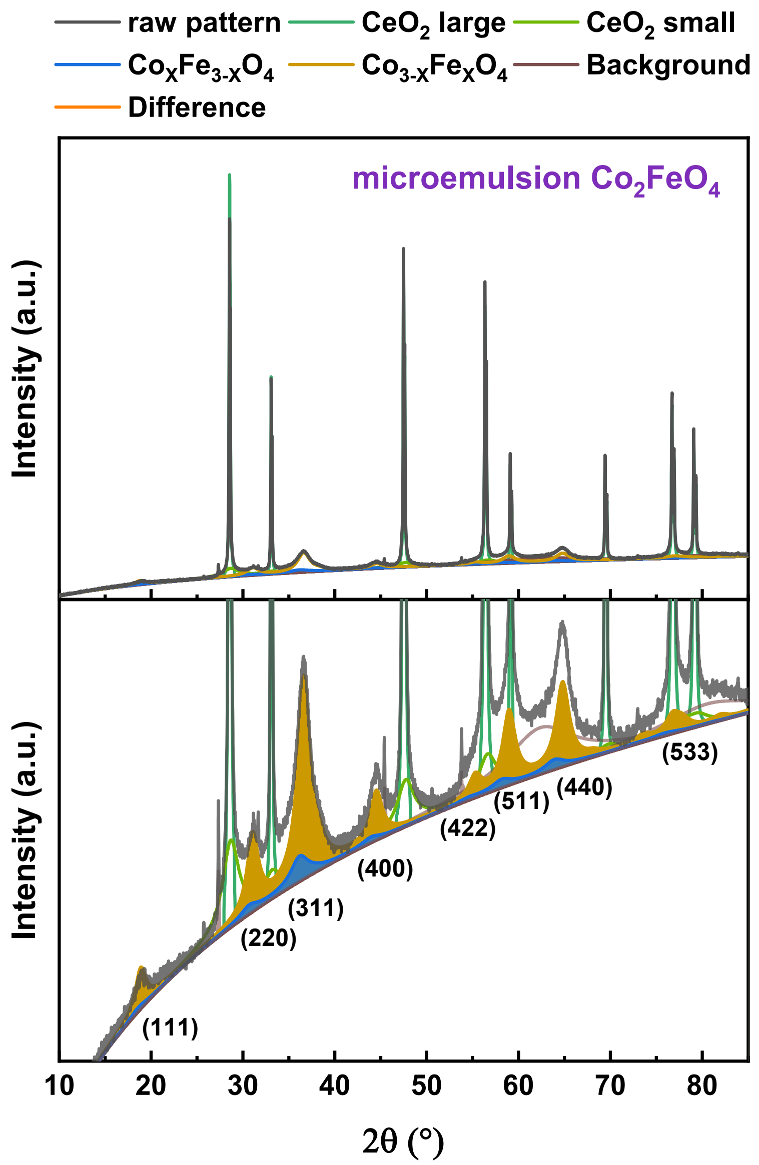

Figure S2. XRD pattern of microemulsion Co_2FeO_4 with CeO_2 NIST-standard and Rietveld refinement. The signal is displayed in black, CeO_2 standard signal from a larger and smaller crystallite is shown in darker green and lime, underlying background signal from the Si substrate is given in brown. The Rietveld refinement was performed considering an Fe-rich spinel phase (Spinel Phase 1) in yellow and a Co-rich spinel phase (Spinel Phase 2) in blue.

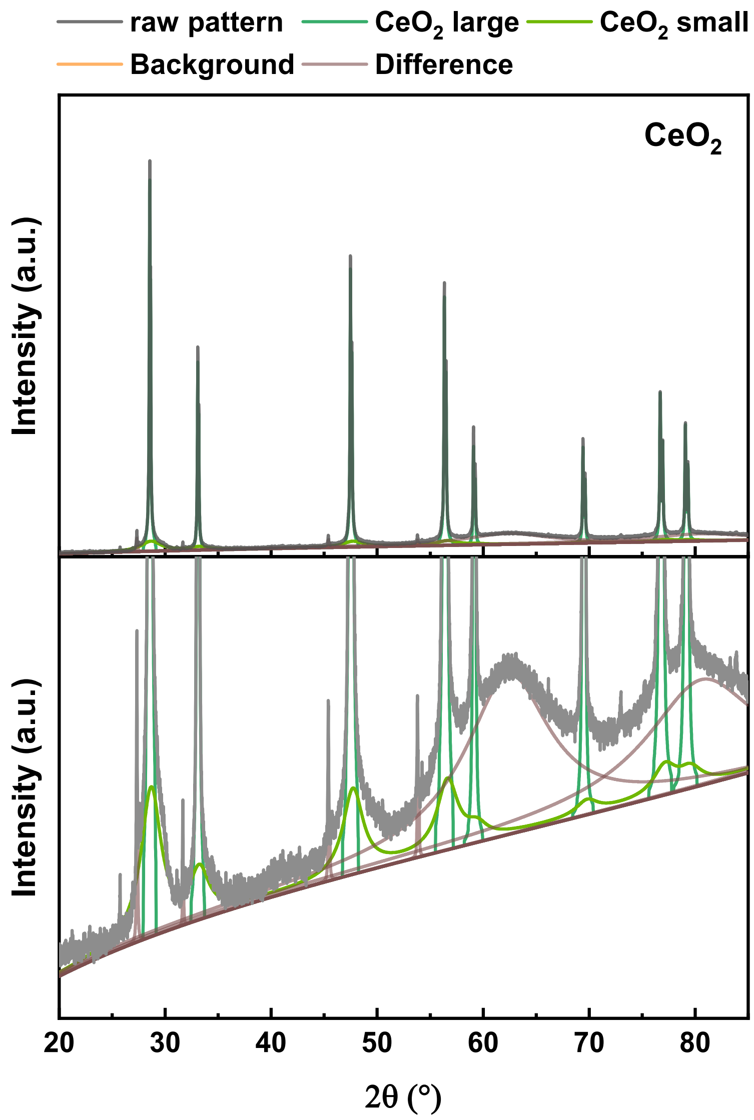

Figure S3. XRD pattern of the CeO_2 NIST-standard and Rietveld refinement. The signal is displayed in black, CeO_2 standard signal from a larger and smaller crystallite is shown in darker green and lime, underlying background signal from the Si substrate is given in brown.

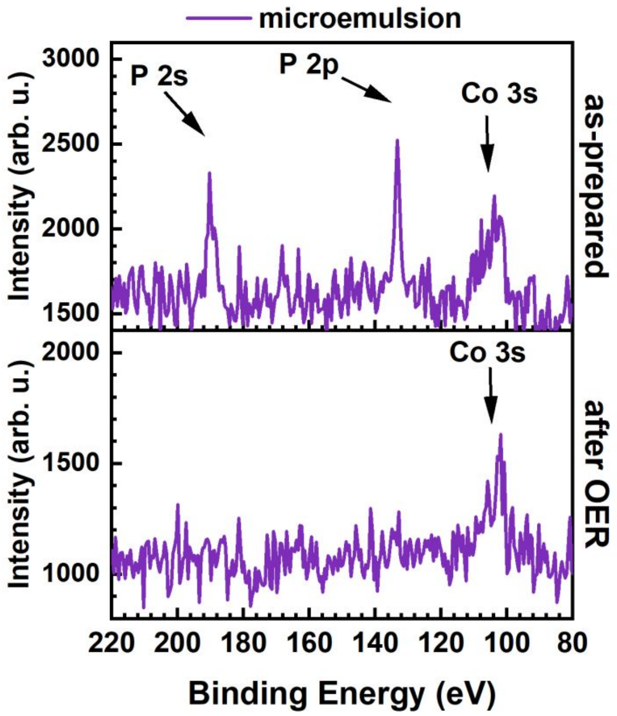

Figure S4. Phosphate region of XPS Survey scan of microemulsion Co_2FeO_4 before and after OER conditioning. The phosphate is completely removed after exposure to electrochemical conditioning.

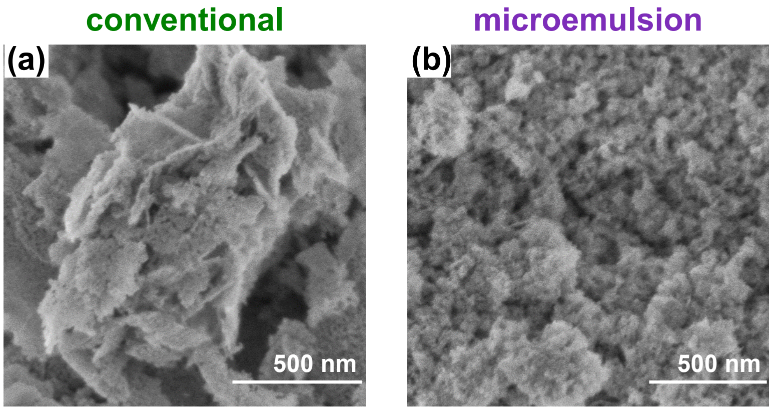

Figure S5. SEM images on glassy carbon of (a) the conventional and (b) the microemulsion Co_2FeO_4 samples in their as-prepared state.

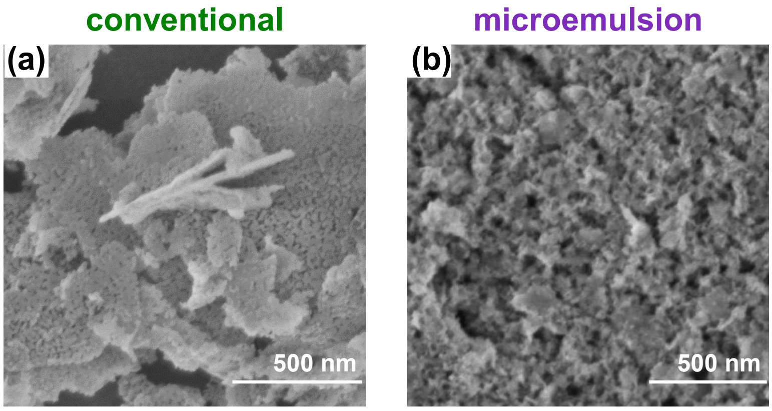

Figure S6. SEM images on glassy carbon of (a) the conventional and (b) the microemulsion Co_2FeO_4 samples after 30 minutes OER at 1.7 V_{RHE}.

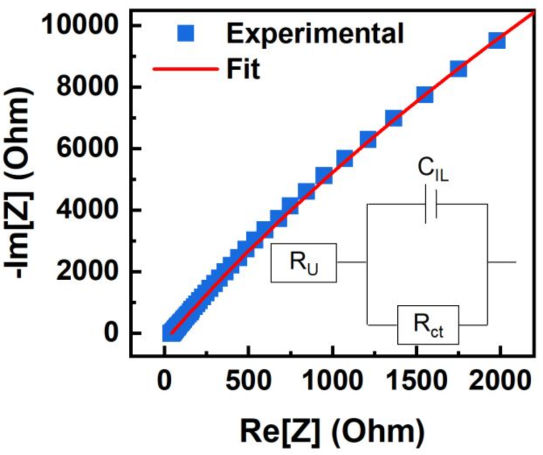

Figure S7. Exemplary showcase of Nyquist plot of microemulsion Co_2FeO_4 at 1 V_{RHE} with no Faradaic current. The uncompensated resistance of the fit equals R_U = 45.41 Ω, double layer capacitance C_{IL} = 14.6 µF and charge-transfer resistance R_{ct} = 790 kΩ.

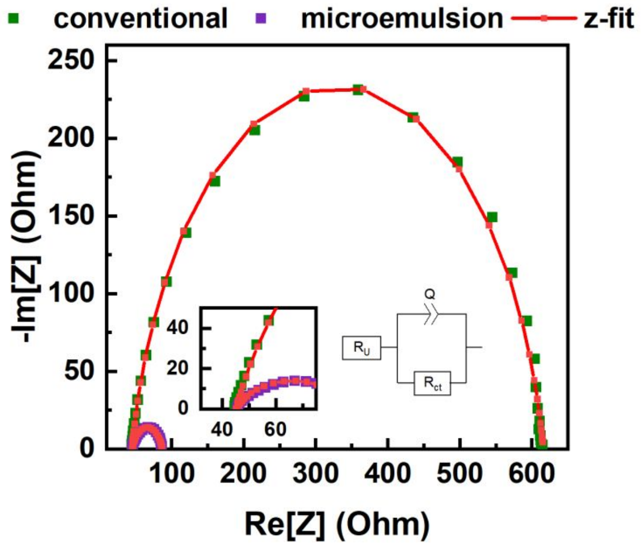

Figure S8. Comparison of Nyquist plots of conventional and microemulsion Co_2FeO_4 samples under OER conditions at ~1.64 V_{RHE}. The circuit consists of the uncompensated solution resistance R_u, the charge transfer resistance R_{ct} and the constant-phase element Q. For the conventional Co_2FeO_4 R_u = 45.11 Ω, double layer capacitance C_{IL} = 10.81 µF and R_{ct} = 570.8 Ω. For the microemulsion Co_2FeO_4, R_u = 45.28 Ω, the double layer capacitance C_{IL} = 15.82 µF and R_{ct} = 41.99 Ω.

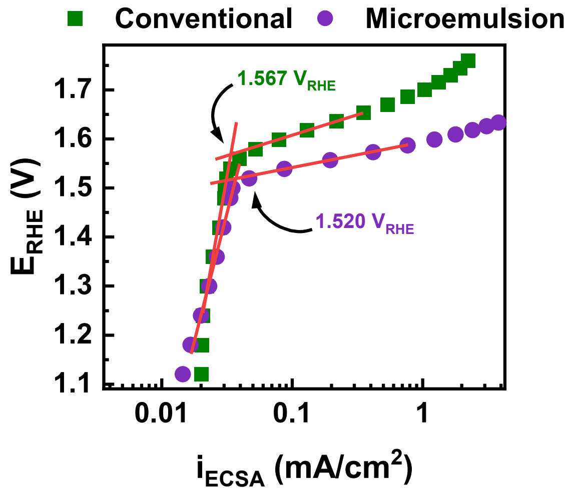

Figure S9. Linear fits of the OER and non-OER regimes of the conventional and microemulsion Co_2FeO_4 samples. The point of intersection of the two linear slopes provides the onset for OER.

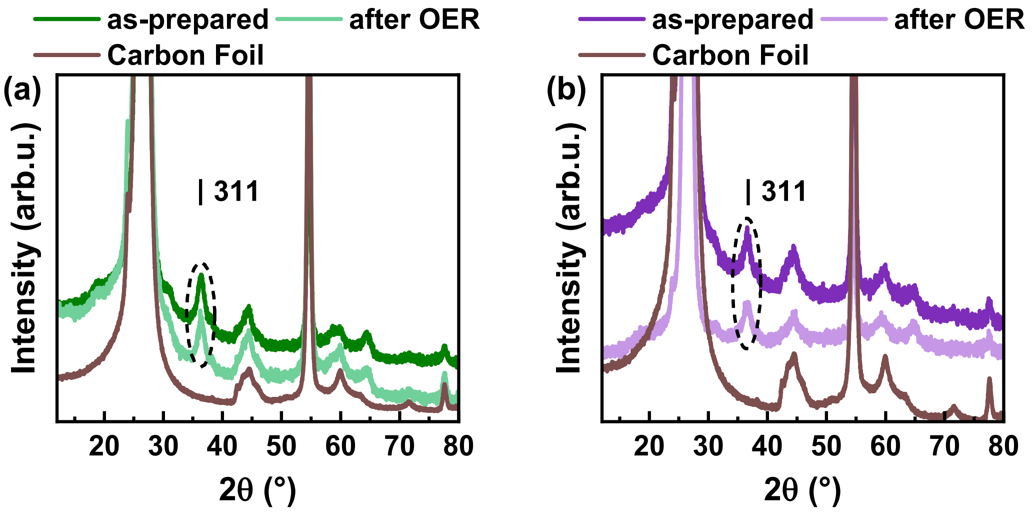

Figure S10. XRD pattern of the conventional (a) and microemulsion (b) Co_2FeO_4 as-prepared and after 30 minutes OER samples. There is an offset between as-prepared, after OER and the carbon foil substrate for a better visualization. The underlying background in brown arises from the carbon foil electrode substrate.

XPS:

Fits of the Co $2p_{3/2}$ based upon multiplet splitting of Co_3O_4 spinels are displayed in Figure S11.[1] The spectra show a Co^{2+} peak at 779.9 eV and a Co^{3+} feature at 781.3 eV. Satellite features of Co^{2+} and Co^{3+} are present at 786.2 and 790.2 eV. The atomic percentage of each species is provided in Table S1. Figure S12 shows the multiplet splitting of the Fe $2p_{3/2}$ main peak. The fits are based on MFe_2O_4 and provide a reasonable fit for Fe^{3+}.[2] Figure S13 displays the fitting of the O 1s peak with the peak fraction in Table S1. The lattice-oxygen is fitted with metal-oxygen and metal-hydroxide peaks at ~529.5 and ~531.2 eV.[3] Carbon-hydroxide and water peaks at ~532.0 and ~533.0 eV originate from the glassy carbon substrate, sample preparation and electrochemistry. Figure S14 displays XPS measurements carried out with a with synchrotron X-ray source and constant kinetic energies of 550 and 200 eV for depth-dependent measurements.

Table S1:

Percentage of the total XPS peak area for different fits of Co-species in the Co $2p_{3/2}$ peak and O 1s peak for conventional and microemulsion Co_2FeO_4 as-prepared (ap) and after OER samples.

**Species/Co_2FeO_4**	**Conventional ap (%)**	**Conventional after OER (%)**	**Microemulsion ap (%)**	**Microemulsion after OER (%)**
**Co $2p_{3/2}$**				
**Co^{2+}**	42.9	39.7	37.9	24.7
**Co^{3+}**	39.0	41.3	36.2	43.0
**sat. Co^{2+}**	13.8	13.9	20.0	16.8
**sat. Co^{3+}**	4.3	5.2	6.0	5.5
**O 1s**				
**M-O**	68.3	54.6	59.6	56.8
**M-OH**	6.8	11.6	22.7	16.4
**C-OH**	21.2	20.7	15.0	20.6
**O-H**	3.7	13.1	2.7	6.2

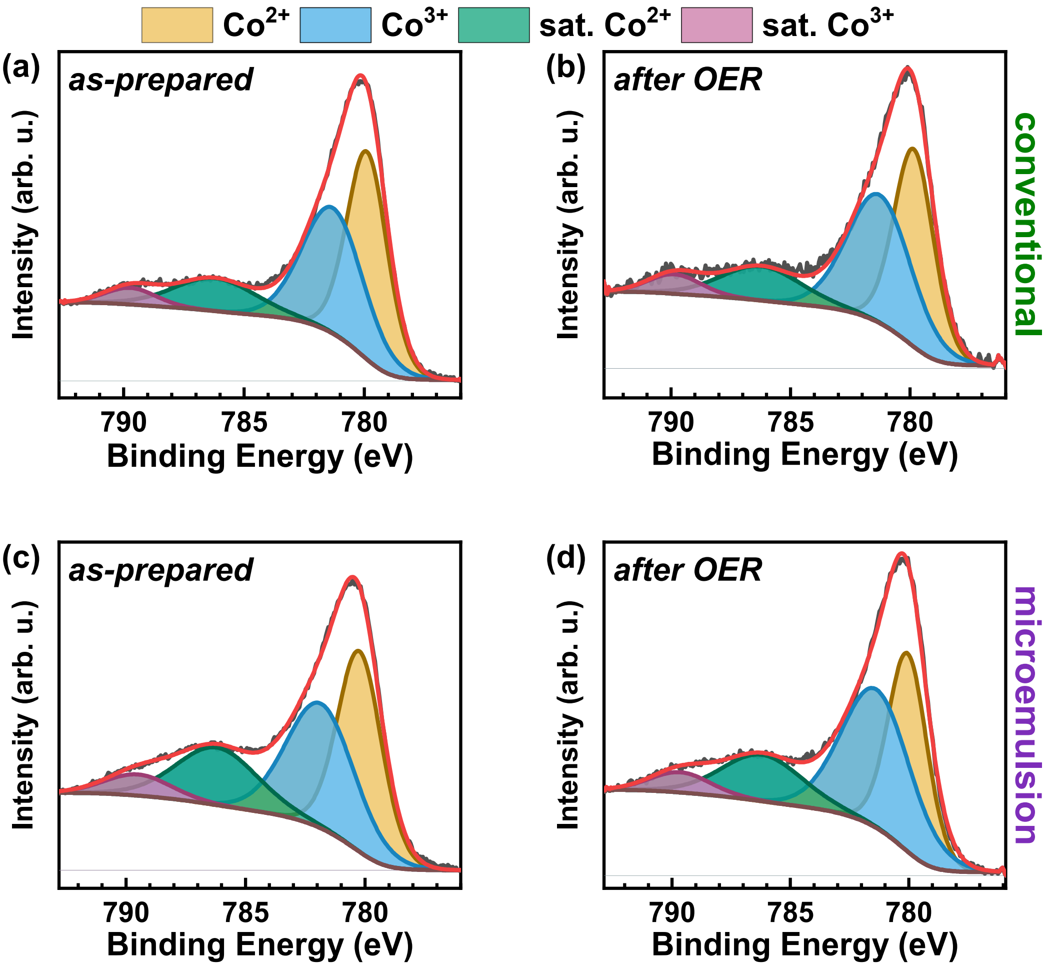

Figure S11. XPS spectra of the Co $2p_{3/2}$ core level region of conventional and microemulsion Co_2FeO_4 samples acquired before and after OER measured without air exposure (*quasi in situ* configuration with an electrochemical cell directly attached to the XPS system). The multiplet fitting is based on suggestions from the literature.[1-2, 4]

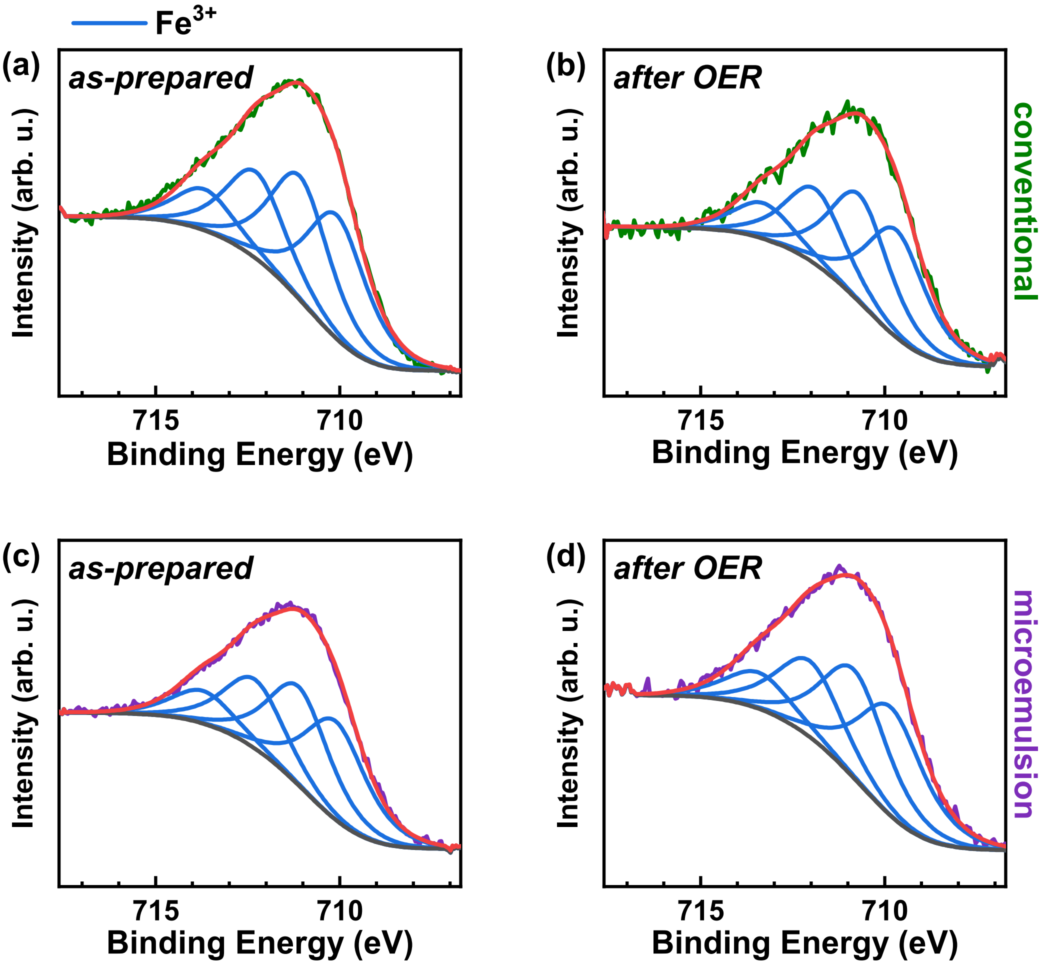

Figure S12. Fe $2p_{3/2}$ core level region of the conventional and microemulsion Co_2FeO_4 samples as-prepared and after OER measured without air exposure (*quasi in situ* XPS configuration with an electrochemical cell directly attached to the XPS system). The fitting approach was adapted for a multiplet structure of Fe^{3+} as reported in the literature for $CoFe_2O_4$ and $NiFe_2O_4$.[1-2, 4]

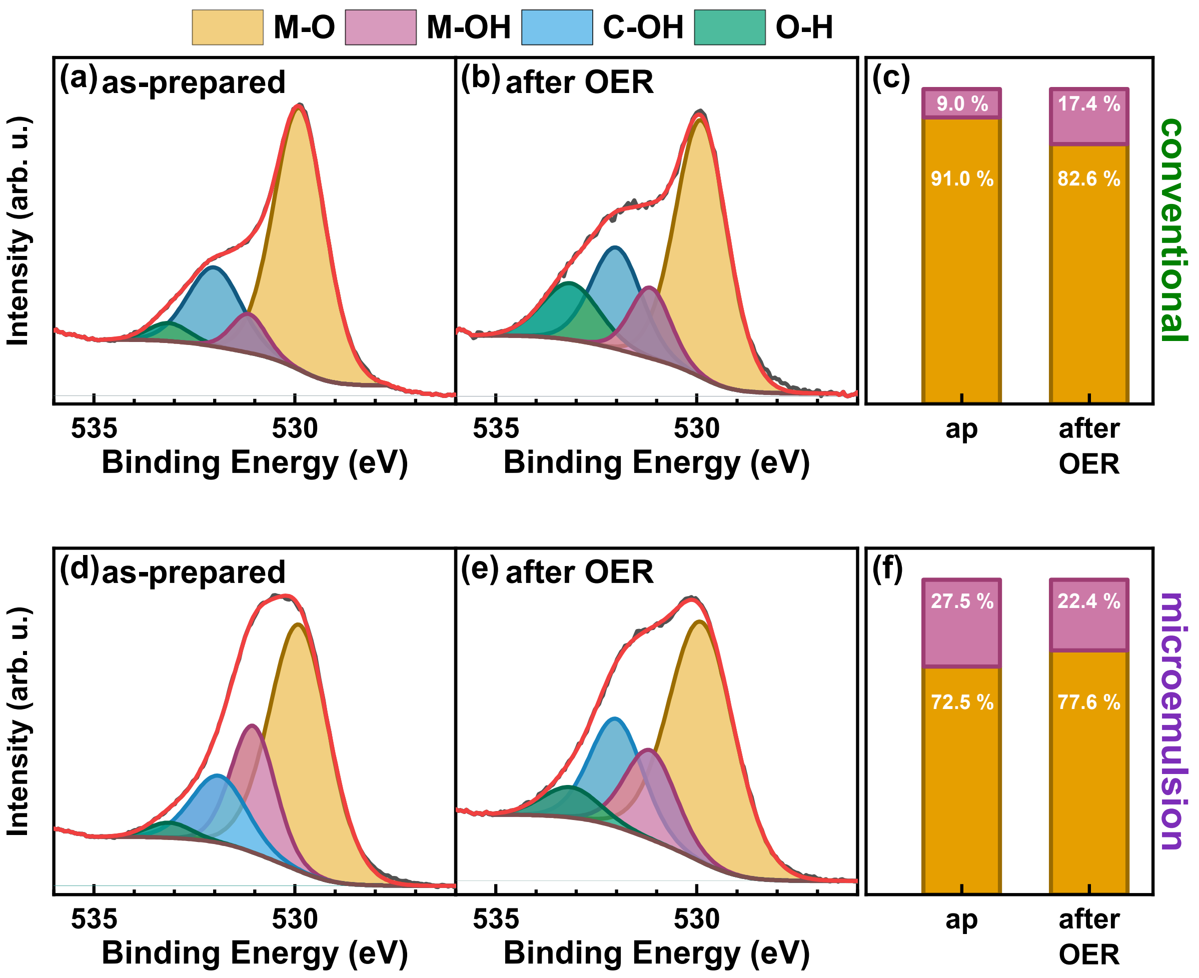

Figure S13. O 1s core level XPS of the conventional and microemulsion Co_2FeO_4 samples as-prepared (ap) and after OER measured with *quasi in situ* XPS. (c) and (f) show the fraction of M-O and M-OH peak area from their total sum of lattice oxygen.

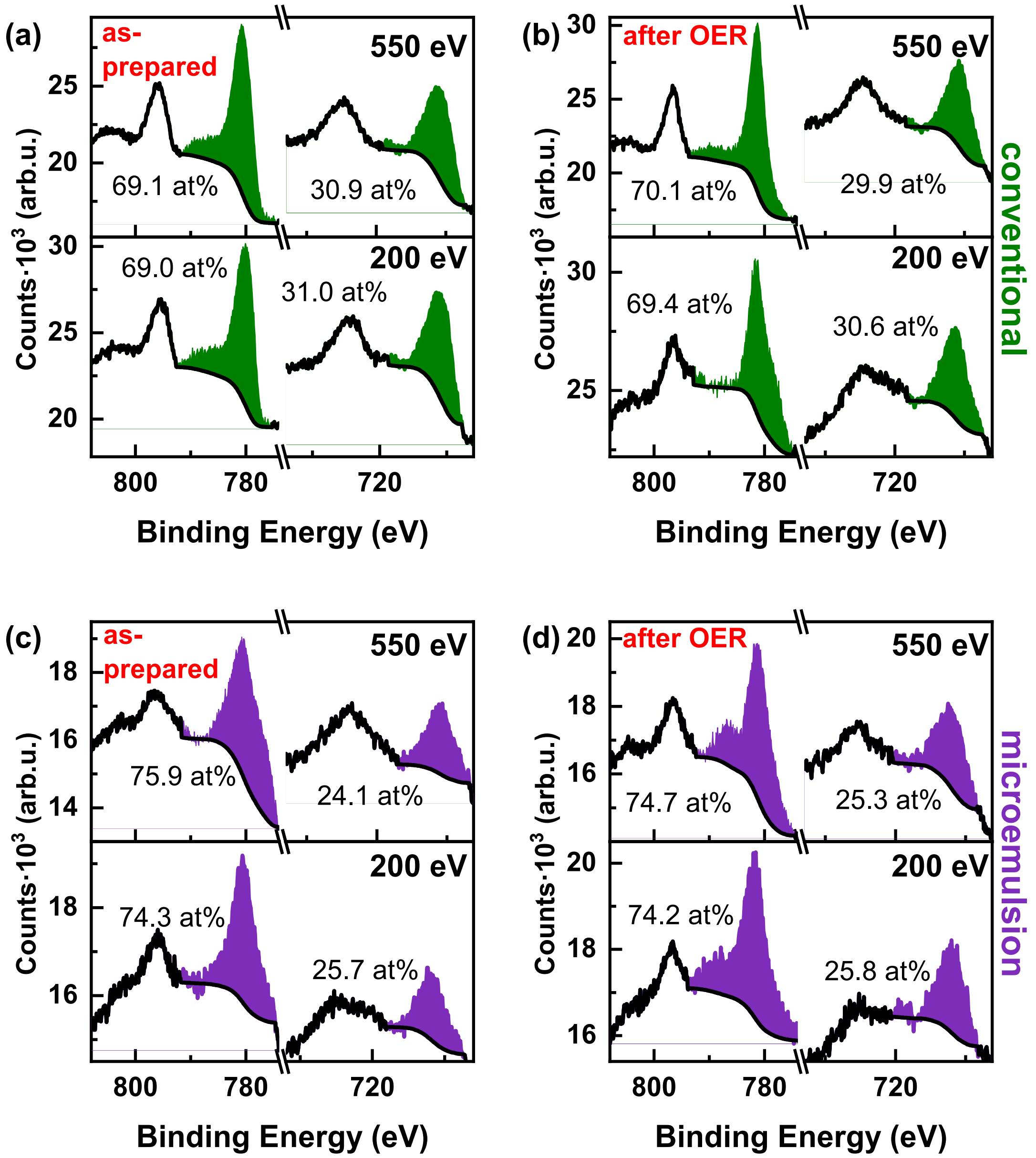

Figure S14. Co and Fe 2p core level regions of conventional and microemulsion Co_2FeO_4 samples as-prepared and after OER with constant kinetic energy. The data were acquired at the ISISS end station of BESSY II synchrotron in Berlin. The excitation energy was chosen such that the photoelectrons of Co and Fe exhibit 550 and 200 eV kinetic energy. The inelastic mean free path was 10.1 Å (Fe) and 10.7 Å (Co) for 550 eV and 5.4 Å (Fe) and 5.8 Å (Co) for 200 eV. The peak area is highlighted in green and purple and was normalized by photon illumination and the photoionization cross-sections.[5] The Co:Fe ratios do not indicate a different composition of the termination layer and the sub-surface and oppose a core-shell structure. The conventional Co_2FeO_4 exhibits the same Co:Fe ratio of 2.2 before and after OER. The microemulsion Co_2FeO_4 sample in the as-prepared state has a surface Co:Fe ratio of 3.0 and 2.9 after OER.

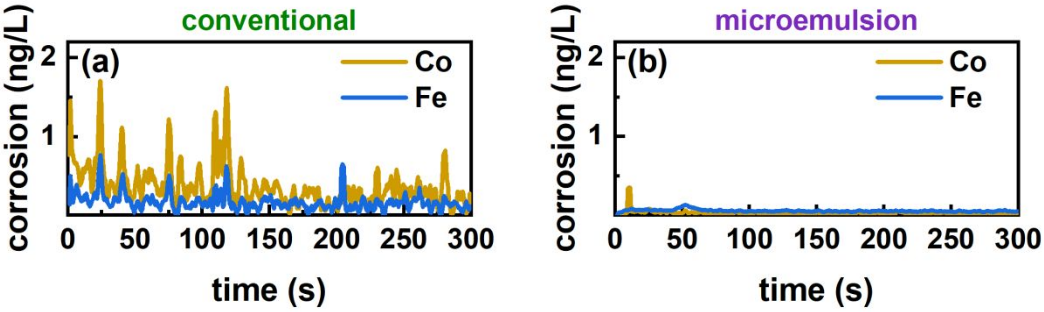

Figure S15. Online ICP-OES Co and Fe corrosion of conventional (a) and microemulsion (b) Co_2FeO_4 at open circuit voltage in ng/L. The conventional catalyst has a dissolution of 0.11 ng_{Co}/min and 0.05 ng_{Fe}/min, the microemulsion catalyst of 0.16 ng_{Co}/min and 0.2 ng_{Fe}/min.

X-ray Absorption Spectroscopy

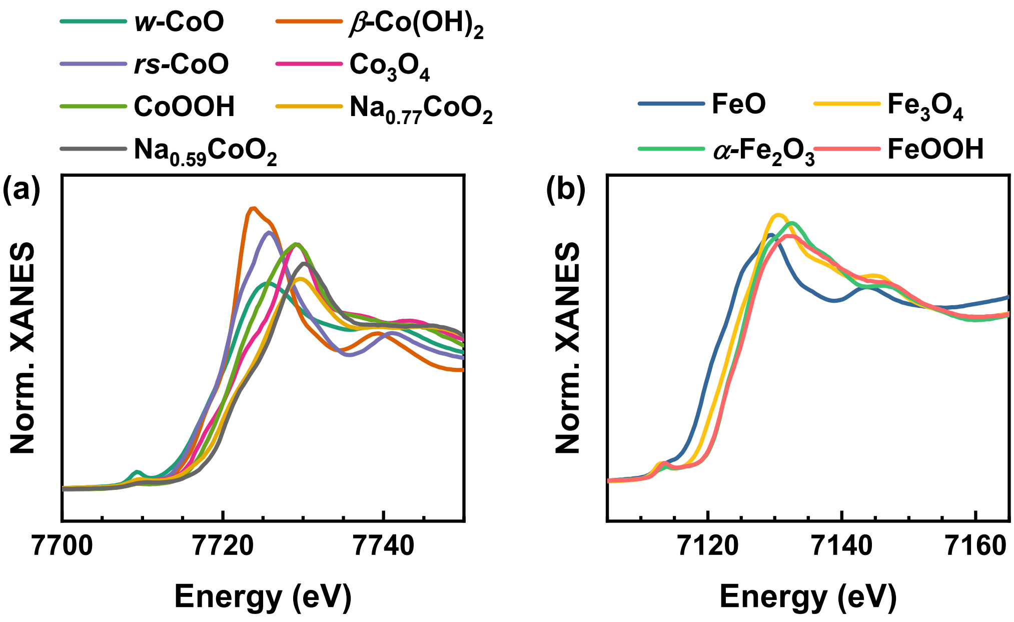

Figure S16. (a) Co K-edge XANES spectra of $CoO_X(OH)_Y$ references for oxidation state calibration and comparison. (b) Fe K-edge XANES spectra for reference compounds used for calibration and comparison.

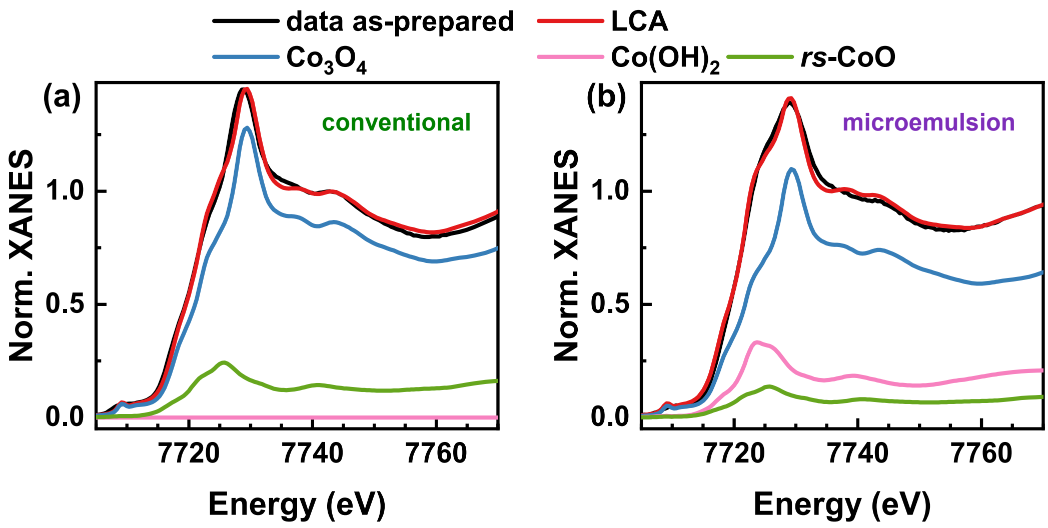

Figure S17. As-prepared XANES Co K-edge spectra of (a) conventional and (b) microemulsion Co_2FeO_4 samples together with a linear combination analysis (LCA). According to these fits, the composition of the samples is: Conventional: 86 % Co_3O_4 + 14 % CoO (+ 0 % $Co(OH)_2$). Microemulsion: 74 % Co_3O_4 + 18 % CoO + 8 % $Co(OH)_2$.

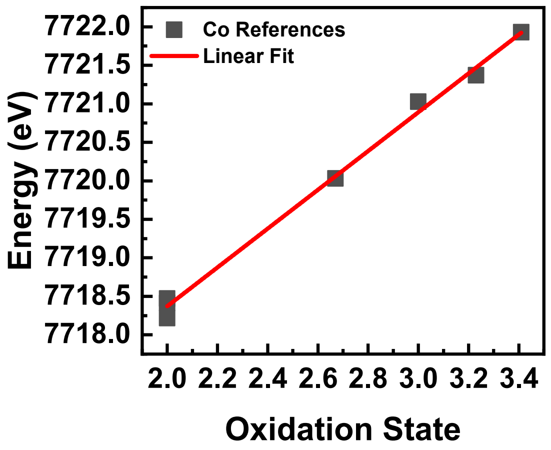

Figure S18. X-ray absorption edge positions at the Co K-edge for Co oxide reference compounds (*rocksalt(rs)*-$Co^{+2}O$, *wurtzite(w)*-$Co^{+2}O$, β-$Co^{+2}(OH)_2$, $Co^{+2.67}{}_3O_4$, CoOOH) with linear calibration 2.511 ± 0.085 eV/Ox.State + 7713.347 ± 0.226 eV.

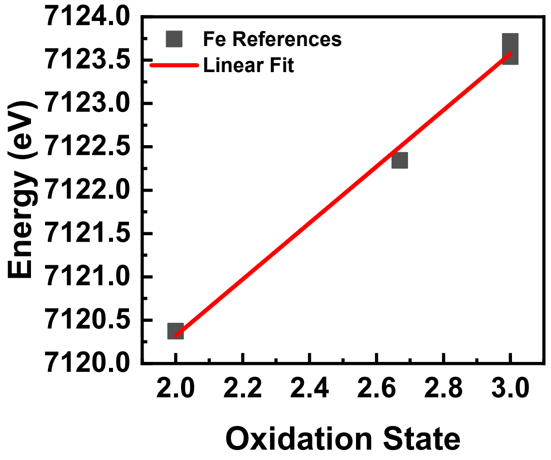

Figure S19. X-ray absorption edge positions at the Fe K-edge for Fe oxide reference compounds (FeO, $Fe^{+2.67}{}_3O_4$, $Fe^{+3}OOH$, $Fe^{+3}{}_2O_3$) with linear calibration 3.254 ± 0.190 eV/Ox.State + 7113.811 ± 0.513 eV.

Table S2:

Oxidation state and absorption edge position in eV for CoO_X and FeO_X references. The edge absorption edge position of the conventional and microemulsion Co_2FeO_4 samples as-prepared, after activation cycling, during OER and after OER for both Co and Fe edge is displayed as well.

Co Edge	#	Oxidation State	Edge Position (eV)
References	*rs*-CoO	2	7718.22
	w-CoO	2	7718.41
	β-$Co(OH)_2$	2	7718.47
	Co_3O_4 (Sigma Aldrich)	2.67	7720.03
	CoOOH	3.0	7721.03
conventional	as-prepared	2.67 ± 0.09	7720.05
	activated	2.79 ± 0.09	7720.33
	at 1.7 V_{RHE} (OER)	2.88 ± 0.09	7720.59
	after OER	2.80 ± 0.09	7720.39
microemulsion	as-prepared	2.58 ± 0.09	7719.83
	activated	2.79 ± 0.09	7720.35
	at 1.7 V_{RHE} (OER)	2.99 ± 0.09	7720.86
	after OER	2.81 ± 0.09	7720.41
**Fe Edge**	**#**	**Oxidation State**	**Edge Position (eV)**
References	FeO	2	7120.37
	Fe_3O_4	2.67	7122.34
	α-Fe_2O_3	3	7123.54
	FeOOH	3	7123.71
conventional	as-prepared	2.91 ± 0.19	7123.27
	activated	2.94 ± 0.19	7123.37
	at 1.7 V_{RHE} (OER)	2.99 ± 0.19	7123.53
	after OER	2.89 ± 0.19	7123.21
microemulsion	as-prepared	2.94 ± 0.19	7123.37
	activated	2.97 ± 0.19	7123.48
	at 1.7 V_{RHE} (OER)	2.99 ± 0.19	7123.54
	after OER	2.92 ± 0.19	7123.31

EXAFS fitting model:

The EXAFS data acquired were fitted by the FEFFIT code. The first three M-O, $M-M_1$ and $M-M_2$ paths were fitted. M-O corresponds to the first coordination shell and the average metal-oxygen contribution. $M-M_1$ represents the metal-metal contribution from an octahedral site with the metal in adjacent octahedral sites. $M-M_2$ describes a total contribution from three non-equivalent paths: (i) path between tetrahedral site and adjacent tetrahedral site, (ii) path between tetrahedral site and adjacent octahedral site and (iii) path between octahedral/tetrahedral sites and adjacent tetrahedral site.

The ratio of total numbers of octahedral and tetrahedral sites in spinel structure is 2:1 (Figure S20), but the distribution of Co and Fe over the available tetrahedral and octahedral sites can vary. To describe it, we introduce a single fitting variable x_{Co} that describes the fraction of Co ions in tetrahedral sites (the ratio of tetrahedrally coordinated Co species and total number of Co ions). To ensure that the 2:1 ratio of Co to Fe is considered in the site occupation, as well as the total ratio of tetrahedral and octahedral sites is preserved, analogous quantity for Fe can be calculated as:

$$x_{Fe} = \frac{\frac{1}{3} - x_{Co}w}{1-w},\#(1)$$

where w = 2/3 is the concentration of Co in the pure spinel sample. For Co_3O_4 and Fe_3O_4 spinel references, x_{Co} and x_{Fe} both are 1/3. For this known ratio, the S_0^2 factors for spinels at the Co and Fe edge were obtained by fitting Co K-edge and Fe K-edge EXAFS spectra of Co_3O_4 and Fe_3O_4. The obtained S_0^2 factors were then used in the analysis of coordination numbers N for catalyst samples.

Knowing the fraction of Co and Fe atoms in the tetrahedral sites x_{Co} and x_{Fe}, the amplitudes $\boldsymbol{A} = S_0^2\, N$ of the main three paths are calculated as follows:

$\boldsymbol{A_{M\text{-}O}}$: $S_0^2\,(6-2x_M)$

$\boldsymbol{A_{M\text{-}M1}}$: $S_0^2\,(6-6x_M)$

$\boldsymbol{A_{M\text{-}M2}}$: $S_0^2\,(6+10x_M)$,

where N is corresponding coordination number and M is Co or Fe.

The XPS measurements provided a larger Co:Fe ratio than the stoichiometric 2:1 ratio, also independent of the kinetic energy, suggesting rather a secondary phase than a core-shell structure. Furthermore, the Co $2p_{3/2}$ and O 1s spectra of the microemulsion catalyst exhibit a significant contribution of CoO and hydroxide, which coincides with a lower average Co oxidation state. Based on this insight, the model to calculate the coordination numbers was further extended to account for the presence of amorphous secondary phase. The concentration of this phase was calculated from the crystallinity measurements provided in Table 1 and was fixed during the fit. We assumed a rock-salt-like structure with a 6-coordinated, environment for the non-spinel Co sites, which is expected to be the dominant structure after calcination at 400 °C.[6] The extended model to quantify the amplitudes for the Co K-edge looked as follows:

$$\boldsymbol{A_{M\text{-}O}}: \quad S_0^2\ ((6-2x_{Co})+6x_C)$$

$$\boldsymbol{A_{M\text{-}M1}}: \quad S_0^2\ (6-6x_{Co})+6x_C)$$

$$\boldsymbol{A_{M\text{-}M2}}: \quad S_0^2\ (6+10x_{Co}),$$

where x_C corresponds to the fraction of crystalline phase. For the conventional Co_2FeO_4 sample the amorphous fraction contributes to 17.58 % (x_x=0.1758) and 37.4 % (x_x=0.374) for the microemulsion Co_2FeO_4. Accordingly, w, the concentration of Co in the sample, was adjusted to 0.62 for conventional and 0.56 for the microemulsion Co_2FeO_4. The Co-metal distances between di-μ-oxo bridged Co-metal sites from Rietveld refinement were used as initial guess of this fitting parameter.

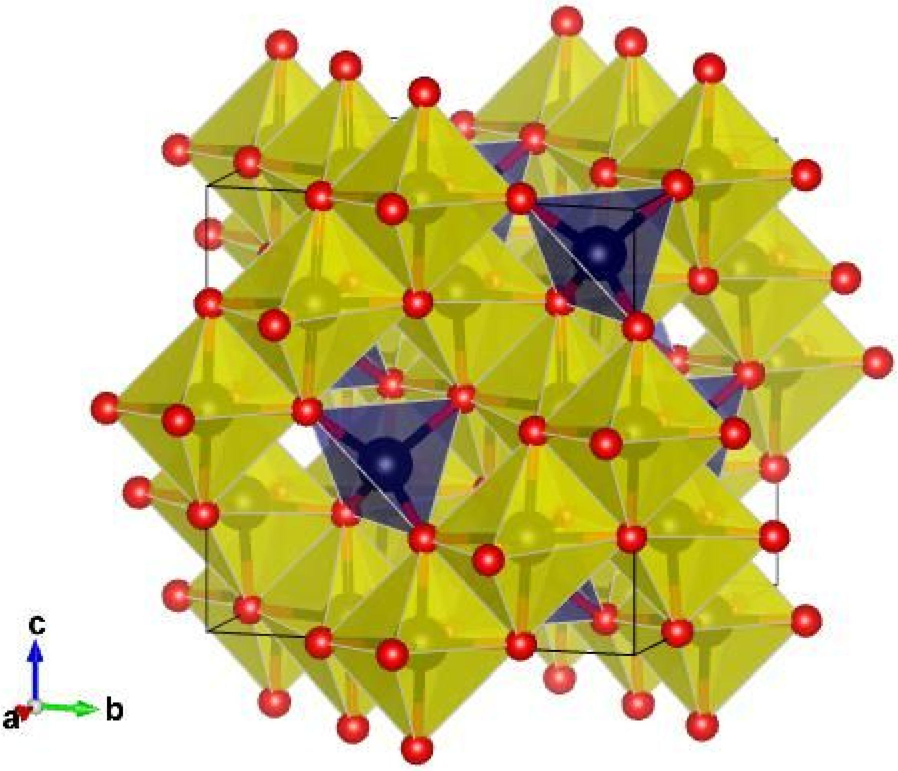

Figure S20. Exemplary spinel model. The octahedrally oriented metal cations are drawn in yellow and tetrahedrally-oriented cations in blue. Two adjacent octahedrally-oriented cations are connected by di-µ-oxo bridges (M-M_1 contribution). Tetrahedrally and octahedrally-oriented cations are connected by mono-µ-oxo bridges (M-M_2 contribution).

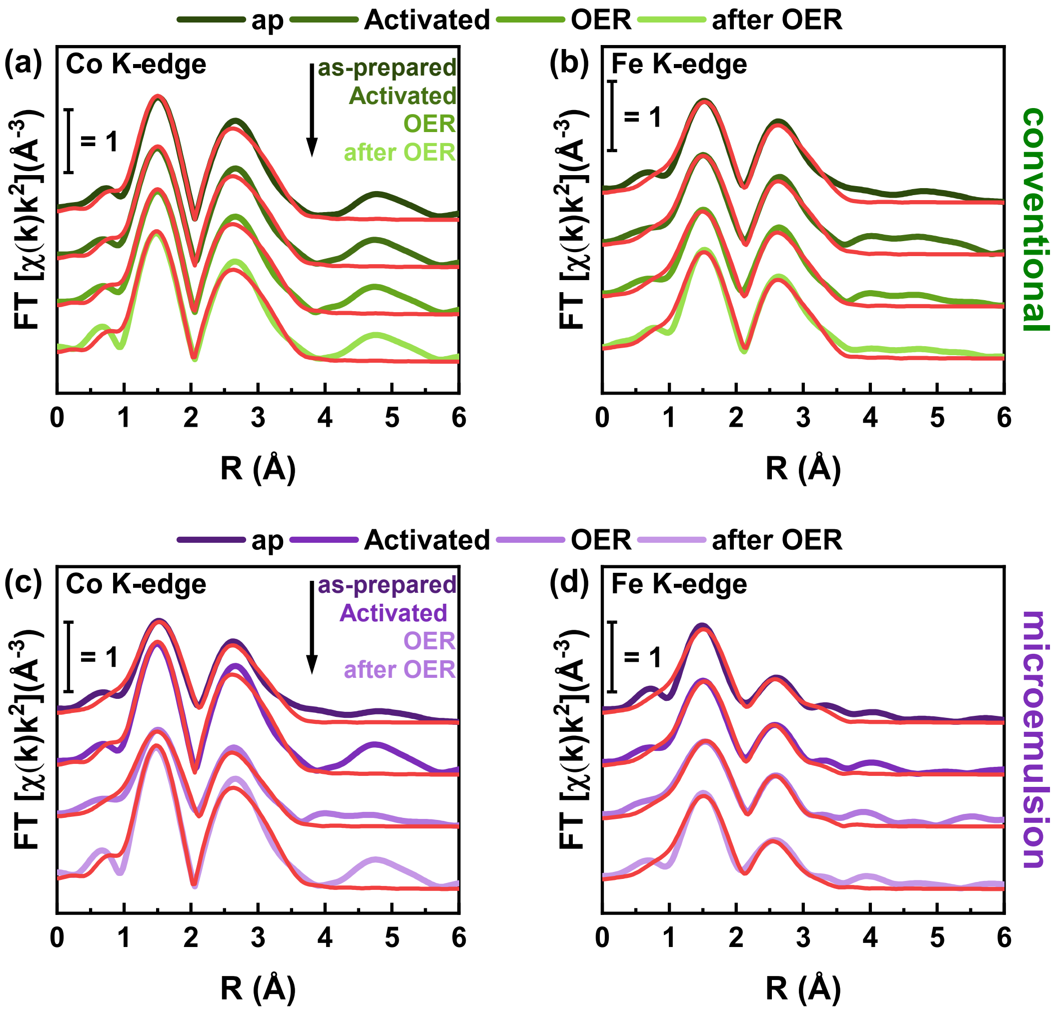

Figure S21. Fourier-transformed EXAFS data measured at the Co and Fe K-edges and their fits (red) for the conventional and microemulsion Co_2FeO_4 samples as-prepared (ap), after electrochemical conditioning (Activated) at 1 V_{RHE}, at 1.7 V_{RHE} (OER) and at 1 V_{RHE} after OER. Each condition was measured for 30 minutes per edge.

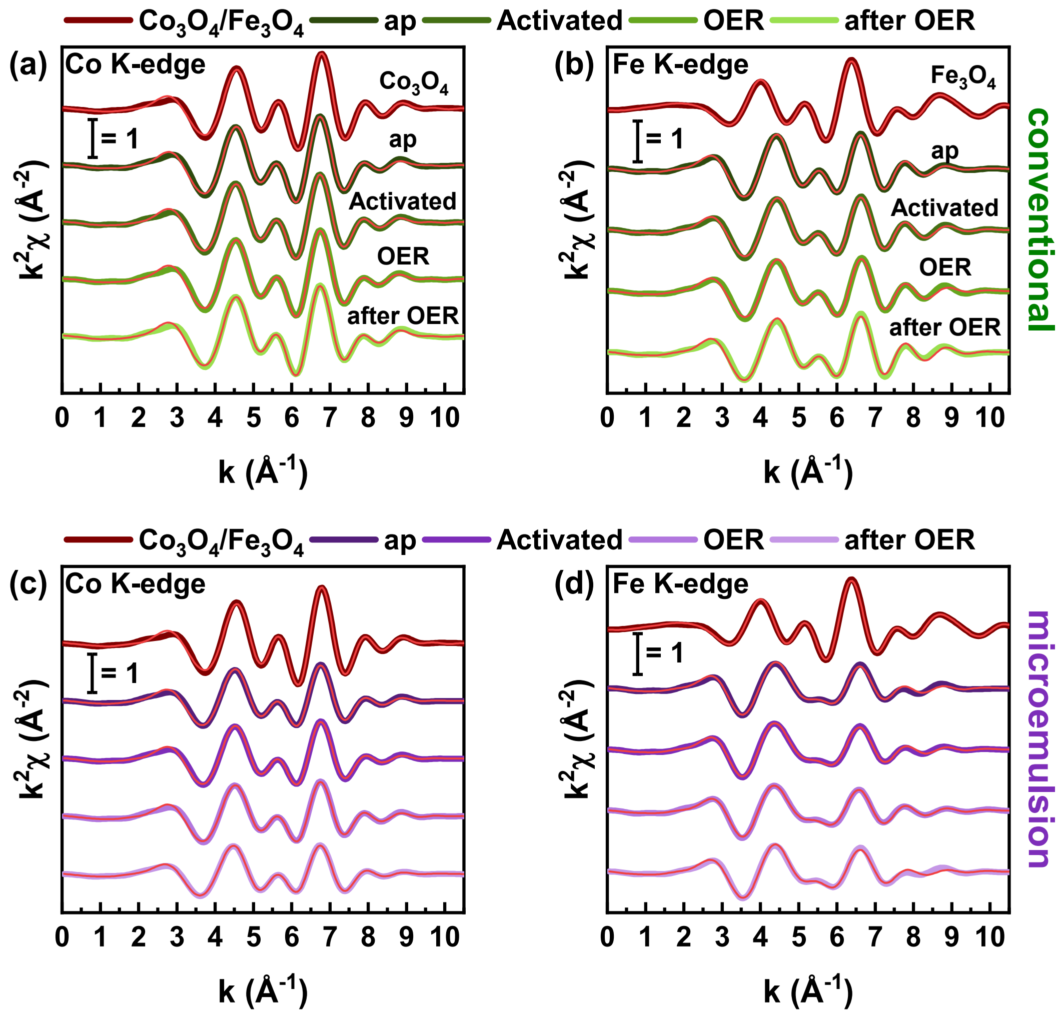

Figure S22. Fourier-filtered EXAFS spectra with fits for conventional and microemulsion Co_2FeO_4 samples as-prepared (ap), after electrochemical conditioning (Activated) at 1 V_{RHE}, at 1.7 V_{RHE} (OER) and at 1 V_{RHE} after OER together with Co_3O_4 and Fe_3O_4 reference samples. Each condition was measured for 30 minutes per edge.

EXAFS fitting parameters:

Coordination numbers ***N***, interatomic distances ***r***, disorder factors $\boldsymbol{\sigma^2}$, energy shifts $\Delta \boldsymbol{E_0}$ and fit quality parameters (***R factor*** in %***)*** extracted from EXAFS fitting of the first three peaks attributed to M-O, $M-M_1$ and $M-M_2$ paths at the Co and Fe K-edges. Uncertainties of the last digit are provided in parentheses. As mentioned above, the variables x_{Co} and x_{Fe} are the fractions of Co and Fe in tetrahedral sites from the total number of Co and Fe ions in the spinel phase. The S_0^2 was 0.75 for the Co edge and 1.0 at the Fe edge.

Table S3:

Co_3O_4 and Fe_3O_4 reference compounds.

**Reference**	**Co/Fe-Path**	**N**	$\mathbf{S_0^2}$	$\mathbf{x_{Co}/x_{Fe}}$	**σ² (Å²)**	**ΔE₀ (eV)**	**r (Å)**	**R (%)**
Co_3O_4	O_1	5.33 (3)	0.75(3)	0.333	0.0030 (6)	0.8(5)	1.907(4)	**1.7**
	Co_1	4.00 (3)			0.0030 (4)		2.850(4)	
	Co_2	9.3 (3)			0.0066 (6)		3.361(5)	
Fe_3O_4	O_1	5.3 (1)	1.0(1)	0.333	0.017 (2)	-2.3(9)	1.98(1)	**1.8**
	Fe_1	4.0 (1)			0.010 (2)		2.98(1)	
	Fe_2	9.3 (1)			0.012 (1)		3.49(1)	

Co Edge:

conventional	Co-Path	N	S_0^2	x_{Co}	σ^2 (Å^2)	ΔE_0 (eV)	r (Å)	R (%)
as-prepared	O_1	5.3 ± 0.6	0.75	0.41 ± 0.06	0.0027± 0.0005	2.2± 0.4	1.918± 0.004	**1.7**
	M_1	4.0 ± 0.8			0.0025± 0.0008		2.870± 0.005	
	M_2	10.1 ± 0.5			0.0072± 0.0007		3.410± 0.006	
Activated	O_1	5.5 ± 0.6	0.75	0.31 ± 0.07	0.0036± 0.0005	2.1± 0.4	1.920± 0.004	**1.7**
	M_1	4.4 ± 0.9			0.0037± 0.0008		2.872± 0.004	
	M_2	9.1 ± 0.6			0.0064± 0.0008		3.407± 0.005	
OER	O_1	5.4 ± 0.7	0.75	0.34 ± 0.07	0.0030± 0.0005	2.0± 0.4	1.915± 0.004	**1.7**
	M_1	4.3 ± 0.9			0.0036± 0.0008		2.865± 0.005	
	M_2	9.4 ± 0.6			0.0061± 0.0008		3.405± 0.006	
after OER	O_1	5.4 ± 0.7	0.75	0.37 ± 0.08	0.0022± 0.0005	1.4± 0.4	1.908± 0.002	**1.8**
	M_1	3.8 ± 0.9			0.0031± 0.0009		2.864± 0.003	
	M_2	9.7 ± 0.7			0.0065± 0.0008		3.400± 0.004	

micro-emulsion	Co-Path	N	S_0^2	x_{Co}	σ^2 (Å^2)	ΔE_0 (eV)	r (Å)	R (%)
as-prepared	O_1	5.4 ± 0.1	0.75	0.48 ± 0.09	0.0064± 0.0005	0.4± 0.4	1.919± 0.004	**1.4**
	M_1	3.6 ± 0.3			0.0071± 0.0009		2.860± 0.005	
	M_2	10.8 ± 0.5			0.0099± 0.0009		3.374± 0.007	
Activated	O_1	5.5 ± 0.1	0.75	0.40 ± 0.07	0.0062± 0.0004	0.8± 0.4	1.919± 0.004	**1.2**
	M_1	4.5 ± 0.3			0.0072± 0.0007		2.859± 0.004	
	M_2	10.0 ± 0.5			0.0089± 0.0009		3.377± 0.006	
OER	O_1	5.6 ± 0.1	0.75	0.34 ± 0.08	0.0077± 0.0005	1.1± 0.4	1.922± 0.004	**1.5**
	M_1	4.8 ± 0.3			0.0086± 0.0008		2.869± 0.005	
	M_2	9.4 ± 0.5			0.008± 0.001		3.380± 0.006	
after OER	O_1	5.5 ± 0.1	0.75	0.39 ± 0.06	0.0089± 0.0004	-0.5± 0.4	1.934± 0.004	**0.6**
	M_1	4.6 ± 0.2			0.0098± 0.0007		2.859± 0.005	
	M_2	9.9 ± 0.4			0.0121± 0.0009		3.366± 0.007	

Fe Edge:

conventional	Fe-Path	N	S_0^2	x_{Fe}	σ^2 (Å^2)	ΔE_0 (eV)	r (Å)	R (%)
as-prepared	O_1	5.6 ± 0.2	1.0	0.20 ± 0.11	0.012± 0.001	2.0± 0.9	1.97± 0.01	**1.3**
	M_1	4.8 ± 0.6			0.011± 0.002		2.94± 0.01	
	M_2	8.0 ± 1.1			0.018± 0.003		3.44± 0.02	
Activated	O_1	5.3 ± 0.2	1.0	0.36 ± 0.12	0.012± 0.002	2±1	1.96± 0.02	**1.3**
	M_1	3.8 ± 0.7			0.008± 0.003		2.94± 0.02	
	M_2	9.6 ± 1.2			0.022± 0.004		3.43± 0.03	
OER	O_1	5.3 ± 0.2	1.0	0.3 ± 0.12	0.013± 0.002	1±1	1.95± 0.02	**1.8**
	M_1	4.0 ± 0.7			0.009± 0.002		2.93± 0.01	
	M_2	9.3 ± 1.2			0.021± 0.004		3.42± 0.03	
after OER	O_1	5.4 ± 0.3	1.0	0.28 ± 0.13	0.011± 0.002	2±1	1.97± 0.02	**2.1**
	M_1	4.3 ± 0.8			0.009± 0.003		2.95± 0.01	
	M_2	8.8 ± 1.3			0.020± 0.004		3.42± 0.03	

micro-emulsion	Fe-Path	N	S_0^2	x_{Fe}	σ^2 (Å^2)	ΔE_0 (eV)	r (Å)	R (%)
as-prepared	O_1	5.7 ± 0.2	1.0	0.15 ± 0.11	0.014± 0.001	1.0± 0.9	1.97± 0.01	**1.5**
	M_1	5.1 ± 0.7			0.017± 0.003		2.98± 0.02	
	M_2	7.5 ± 1.1			0.027± 0.006		3.37± 0.03	
Activated	O_1	5.5 ± 0.2	1.0	0.25 ± 0.09	0.013± 0.001	2±1	1.98± 0.01	**0.6**
	M_1	4.5 ± 0.6			0.013± 0.002		2.96± 0.01	
	M_2	8.5 ± 0.9			0.043± 0.015		3.34± 0.07	
OER	O_1	5.3 ± 0.2	1.0	0.33 ± 0.10	0.014± 0.001	2.7± 0.9	1.99± 0.01	**0.6**
	M_1	4.0 ± 0.6			0.011± 0.002		2.97± 0.01	
	M_2	9.3 ± 1.0			0.05± 0.02		3.34± 0.07	
after OER	O_1	5.5 ± 0.2	1.0	0.27 ± 0.08	0.0133± 0.0008	0.9± 0.6	1.967± 0.007	**1.3**
	M_1	4.4 ± 0.5			0.014± 0.002		2.950± 0.008	
	M_2	8.7 ± 0.8			0.041± 0.008		3.40± 0.04	

References

(1) Biesinger, M. C.; Payne, B. P.; Grosvenor, A. P.; Lau, L. W.; Gerson, A. R.; Smart, R. S. C., Resolving surface chemical states in XPS analysis of first row transition metals, oxides and hydroxides: Cr, Mn, Fe, Co and Ni. *Appl. Surf. Sci.* **2011,** *257* (7), 2717-2730.
(2) Gupta, R. P.; Sen, S. K., Calculation of multiplet structure of core p-vacancy levels. *Phys. Rev. B* **1974,** *10* (1), 71-77.
(3) Yang, J.; Liu, H.; Martens, W. N.; Frost, R. L., Synthesis and characterization of cobalt hydroxide, cobalt oxyhydroxide, and cobalt oxide nanodiscs. *J. Phys. Chem. C* **2010,** *114* (1), 111-119.
(4) Fantauzzi, M.; Secci, F.; Angotzi, M. S.; Passiu, C.; Cannas, C.; Rossi, A., Nanostructured spinel cobalt ferrites: Fe and Co chemical state, cation distribution and size effects by X-ray photoelectron spectroscopy. *RSC Adv.* **2019,** *9* (33), 19171-19179.
(5) Yeh, J.; Lindau, I., Atomic subshell photoionization cross sections and asymmetry parameters: $1 \leqslant Z \leqslant 103$. *At. Data Nucl.* **1985,** *32* (1), 1-155.
(6) Nam, K. M.; Shim, J. H.; Han, D.-W.; Kwon, H. S.; Kang, Y.-M.; Li, Y.; Song, H.; Seo, W. S.; Park, J. T., Syntheses and characterization of wurtzite CoO, rocksalt CoO, and spinel Co_3O_4 nanocrystals: their interconversion and tuning of phase and morphology. *Chem. Mater.* **2010,** *22* (15), 4446-4454.
